# Optimal concentration of ropivacaine for brachial plexus blocks in adult patients undergoing upper limb surgeries: a systematic review and meta-analysis

**DOI:** 10.3389/fphar.2023.1288697

**Published:** 2023-11-16

**Authors:** Lin Wu, Weiyi Zhang, Xiangdong Zhang, Yinglong Wu, Hua Qu, Donghang Zhang, Yiyong Wei

**Affiliations:** ^1^ Department of Anesthesiology, West China Hospital, Sichuan University, Chengdu, China; ^2^ Laboratory of Anesthesia and Critical Care Medicine, National-Local Joint Engineering Research Centre of Translational Medicine of Anesthesiology, West China Hospital of Sichuan University, Chengdu, China; ^3^ Department of Anesthesiology, First People’s Hospital of Tianshui City, Tianshui, China; ^4^ Department of Anesthesiology, Pu’er People’s Hospital, Pu'er, China; ^5^ Department of Anesthesiology, Longgang District Maternity & Child Healthcare Hospital of Shenzhen City (Longgang Maternity and Child Institute of Shantou University Medical College), Shenzhen, China

**Keywords:** brachial plexus block, concentration, meta-analysis, nerve block, ropivacaine, systematic review, upper limb surgery

## Abstract

**Aim of the Study:** Brachial plexus block (BPB) is widely used for patients undergoing upper limb surgeries. Ropivacaine is the most commonly used local anesthetic for BPB. This study aimed to identify the optimal ropivacaine concentration for BPB in adult patients undergoing upper limb surgeries.

**Materials and Methods:** PubMed, Embase, the Cochrane Library, and Web of Science were searched to identify randomized controlled trials (RCTs) that compared the effects of different concentrations of ropivacaine for BPB in adult patients undergoing upper limb surgeries. The primary outcomes were the onset time of sensory and motor block. RevMan 5.4 software was used for analysis. The GRADE approach was used to assess evidence quality.

**Results:** Nine studies involving 504 patients were included. Compared to 0.5% ropivacaine, 0.75% ropivacaine shortened the onset time of sensory (WMD, −2.54; 95% CI; −4.84 to −0.24; <0.0001, moderate quality of evidence) and motor blockade (WMD, −2.46; 95% CI, −4.26 to −0.66; *p* = 0.01; moderate quality of evidence). However, 0.5% and 0.75% ropivacaine provided similar duration time of sensory (WMD, −0.07; 95% CI, −0.88 to 0.74; *p* = 0.81; high quality of evidence) and motor blockade (WMD, −0.24; 95% CI, −1.12 to 0.65; *p* = 0.55; high quality of evidence), as well as time to first request for oral analgesia (WMD, −1.57; 95% CI, −3.14 to 0.01; *p* = 0.5; moderate quality of evidence).

**Conclusion:** Moderate-quality evidence suggested that, in terms of the onset time of sensory and motor blockade, 0.75% ropivacaine is a preferred concentration for BPB in upper limb surgeries.

**Systematic Review Registration:** identifier CRD42023392145.

## 1 Introduction

Peripheral nerve blocks (PNBs) can often replace general anesthesia or provide a good complement by reducing the consumption of general anesthetics and opioid-related complications (Aisling et al., 2022; Jiao et al., 2022; Li et al., 2022). In addition, PNBs do not interfere with the function of autonomic nerves and can provide both satisfactory surgical conditions and prolonged postoperative analgesia, with the advantages of safety, satisfaction, and rapid postoperative recovery (Chan et al., 2001; Long et al., 2002; Hadzic et al., 2005). In clinical work, the onset and duration time of local anesthetics are often used as a reference index for anesthesiologists to select an appropriate anesthesia program to achieve satisfactory blocking effects (Safa et al., 2021; Wang et al., 2023). The onset time of local anesthetics mainly depends on their concentration and dose and whether vasoconstricting drugs are used (Ranganath et al., 2022). The rapid onset time of nerve block can quickly reduce the discomfort of patients, eliminate abnormal sensation, help reduce movement interference and pain interference during surgery, and effectively reduce the use of intraoperative opioids (Long et al., 2002; Dai and Huo, 2023). The duration of a single nerve block is another key factor affecting the analgesic effect after regional anesthesia (Fredrickson et al., 2012). A longer block duration can prolong postoperative analgesia, reduce postoperative pain, opioid consumption and related side effects, and improve patient satisfaction (Fredrickson et al., 2012; Safa et al., 2021). Therefore, the onset and duration time of the block are two important indicators to evaluate the effects of PNBs.

For upper limb surgeries, brachial plexus block (BPB) is the preferred anesthetic option (Kalthoff et al., 2022; Mojica et al., 2022; Kang and Ko, 2023). Currently, many types of local anesthetics have been used for BPB, such as lidocaine, bupivacaine, and ropivacaine (Hughes et al., 2013). However, the most widely used local anesthetic for BPB is ropivacaine, which is safer due to its lower central nervous system and cardiac toxicity (Vainionpää et al., 1995; Marhofer et al., 1998; McClellan and Faulds, 2000). Furthermore, as a long-acting local anesthetic, ropivacaine is superior to medium-acting lidocaine in providing longer analgesia (Vainionpää et al., 1995; Marhofer et al., 1998; McClellan and Faulds, 2000). However, the clinical concentrations of ropivacaine used for BPB vary from 0.25% to 1% (Furutani et al., 2021; Ran et al., 2022; Xu et al., 2022). Many randomized controlled trials (RCTs) have compared the clinical effectiveness and safety between different concentrations of ropivacaine for BPB in upper limb surgeries; however, the optimal concentration remains controversial (Markham et al., 1996; Marhofer et al., 1998; McClellan and Faulds, 2000). Therefore, we performed this meta-analysis to determine the optimal concentration of ropivacaine for BPB in upper limb surgeries, which will provide evidence for the concentration selection of ropivacaine in BPB.

## 2 Materials and methods

### 2.1 Search strategies

This study was conducted based on the Preferred Reporting Items for Systematic Reviews and Meta-Analyses (PRISMA) guidelines (Moher et al., 2009), and the study protocol was registered in the prospective register of systematic reviews (PROSPERO number: CRD42023392145). To find suitable studies for inclusion, we searched the PubMed, Embase, Cochrane Library, and Web of Science databases from their inception to 6 December 2022. A combination of subject and free terms was used, including ‘Brachial Plexus Block’, brachial plexus anesthesia’, ‘ropivacaine’, and ‘concentration’. A detailed search strategy was shown in Supplementary Material S1.

### 2.2 Study selection

Two authors (L.W. and D.Z.) independently identified eligible studies by reading the titles and abstracts of the initially included studies. The full texts of potentially relevant articles were retrieved for final inclusion. Contradictions were resolved through discussion with another author (W.Z.).

### 2.3 Inclusion and exclusion criteria

Studies were eligible if (1) they were RCTs in English; (2); the study population was adults (≥18 years old) undergoing upper limb surgeries; (3); they compared 0.5% ropivacaine (as the control group) with other concentrations of ropivacaine for brachial plexus blocks; and (4) they reported any of the consensus-based primary and secondary outcomes. Trials that did not meet the inclusion criteria were excluded.

### 2.4 Primary and secondary outcome indicators

The primary outcomes included the onset time of sensory and motor blockade (minutes). The secondary outcomes evaluated the duration of sensory and motor blockade (hours) and the time to first request for oral analgesia (hours).

### 2.5 Data extraction

Research information from the eligible RCTs was independently extracted by two researchers (L.W. and D.Z.) using a standardized information extraction form agreed upon in advance, which included the study title, publication date, first author, geographical location, patient characteristics, study design, type of surgery, interventions and control groups, use of nerve blocks, and outcomes.

### 2.6 Risk of bias assessment

The risk of bias for the included studies was evaluated using the Risk of Bias Assessment Tool ROB2 (revised version 2019) for RCTs recommended by the Cochrane Handbook. This tool includes five areas of bias: randomization process, deviations from intended interventions, missing outcome data, selection of the reported result and measurement of the outcome. For each section, studies were classified as having a low, some concerns, or high risk of bias. Risk of bias evaluations were performed independently by two evaluators, and the results were cross-checked. Disagreements were resolved with another author (W.Z.).

### 2.7 GRADE

The quality of evidence for the results of the main outcomes was graded using the GRADE system (Guyatt et al., 2008), focusing on the following five factors: (1): risk of bias, (2), inconsistency, (3), indirectivity, (4), inaccuracy, and (5) publication bias. The quality of the GRADE evidence ranged from high to very low.

### 2.8 Statistical analyses

All analysis were conducted using RevMan 5.4. Continuous variables were summarized as the mean difference (MD) with 95% confidence intervals (CI) to present data, while dichotomous data were calculated by risk ratios (RR) with 95% CI. Data that were reported as the median and range were converted into the mean ± SD using the methods described by Wan et al. (Wan et al., 2014) and Hozo et al. (Hozo et al., 2005). The heterogeneity of the included studies was analyzed using the chi-square test and evaluated in conjunction with I^2^ statistics. A random-effects model was used due to the potentially high methodological and clinical heterogeneity. Given that brachial plexus block has different approaches (interscalene, costoclavicular space, supraclavicular, and axillary), we conducted subgroup analyses to ensure consistency of the results. Sensitivity analysis was performed to show the effect of a single study on the overall heterogeneity. *p* < 0.05 was considered statistically significant.

## 3 Results

### 3.1 Screening process for study inclusion

**FIGURE 1:**
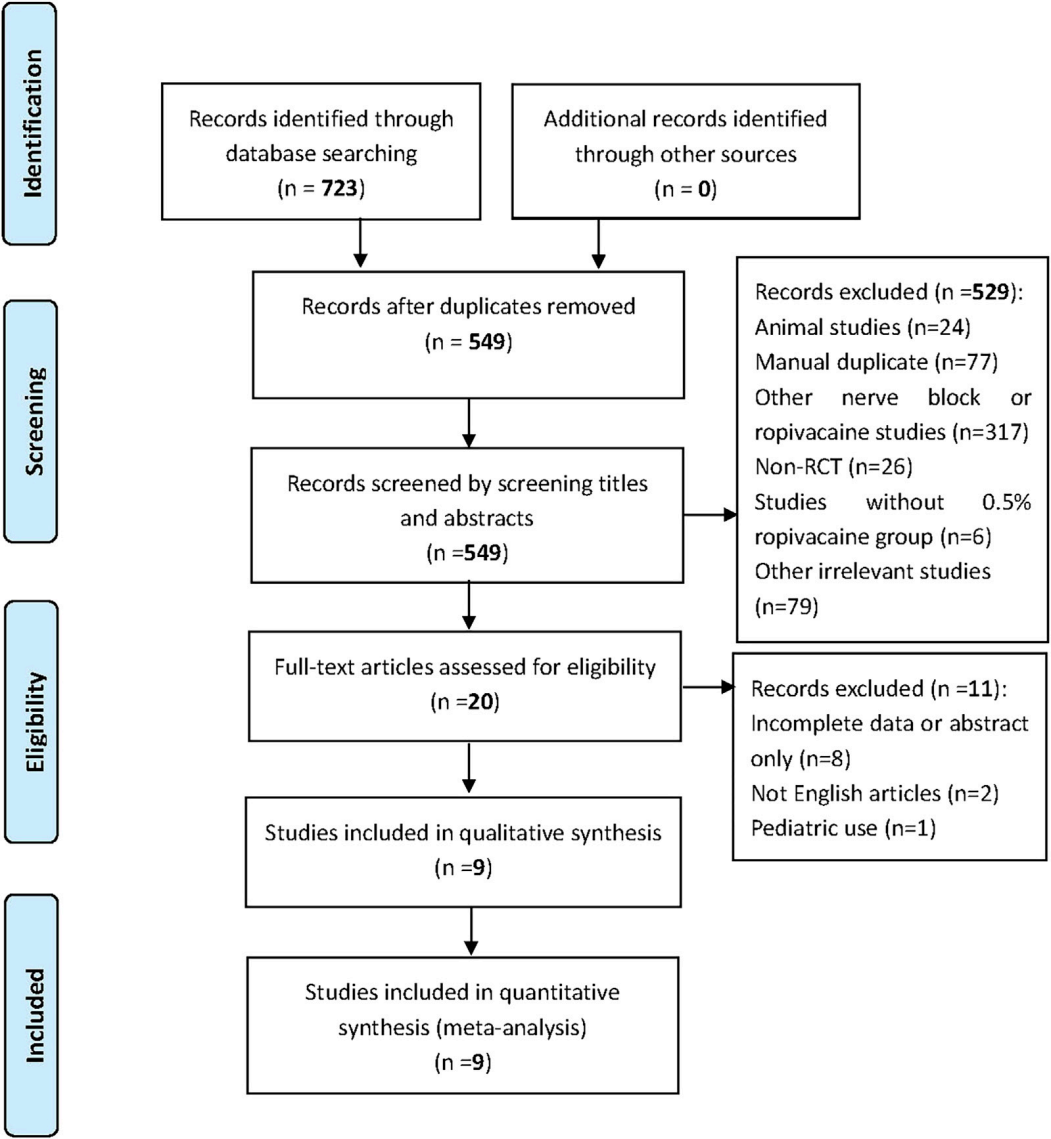
Flow diagram of study selection.

The initial search of the four databases yielded 723 documents for possible inclusion. After the removal of duplicates by Endnote, the titles and abstracts of the remaining 549 articles were reviewed. Then, the full texts of 20 potentially relevant studies were read. Finally, nine RCTs published between 1999 and 2022 were included in this meta-analysis (Klein et al., 1998; Casati et al., 1999a; Casati et al., 1999b; Bertini et al., 1999; Krone et al., 2001; Venkatesh et al., 2016; Wenwen et al., 2016; Safa et al., 2021; Wang et al., 2023). A detailed flowchart of the study selection process was shown in Figure 1.

### 3.2 Study characteristics and bias

**FIGURE 2:**
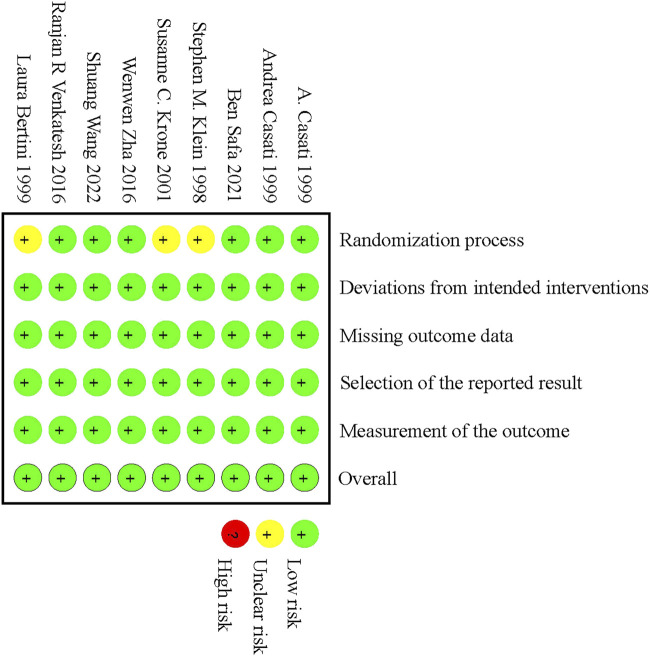
Risk of bias summary.

The baseline characteristics of the nine RCTs included in the study were summarized in Table 1. The nine RCTs included 504 patients who underwent upper limb surgeries after BPB using different concentrations of ropivacaine. Of these 9 studies, three were from Asia (China and India) (Venkatesh et al., 2016; Wenwen et al., 2016; Wang et al., 2023), three were from Europe (Italy) (Casati et al., 1999a; Casati et al., 1999b; Bertini et al., 1999), and three were from North America (United States of America and Canada) (Klein et al., 1998; Krone et al., 2001; Safa et al., 2021). The patients’ ages ranged from 18 to 80 years. According to the Cochrane Risk of Bias Assessment Tool, most aspects showed a low risk of bias, with the exception of three studies (Klein et al., 1998; Bertini et al., 1999; Krone et al., 2001) that demonstrated an unclear risk of bias in the randomization process. The risk of bias of the included studies was shown in Figure 2 and Supplementary Material S2. Publication bias was not assessed using funnel plots because the number of included studies for each outcome indicator was <10.

**TABLE 1:** Characteristics of included studies.

Study	Country	Sample	Age (yr)	ASA	Surgical procedure	BPB approach	BPB	Injection volume	Anesthesia	Outcomes
Casati et al. (1999a)	Italy	45	18–65	I-II	elective shoulder surgery	Interscalene	Ropivacaine 0.5% (n = 15), 0.75% (n = 15), 1% (n = 15)	20 mL	NR	a, b, c, d, e
Casati et al. (1999b)	Italy	20	18–65	I-II	elective shoulder surgery	Interscalene	Ropivacaine 0.5% (n = 10), 0.75% (n = 10)	20 mL	None	a, b, d, e, f, g
Safa et al. (2021)	Canada	40	18–80	I-III	arthroscopic shoulder surgery	Interscalene	Ropivacaine 0.5% (n = 20), 1% (n = 20)	5 mL	GA	c, e, h, i, j, k, l
Klein et al. (1998)	North Carolina (United States of America)	50	≥18	I-III	outpatient shoulder surgery	Interscalene	Ropivacaine 0.5% (n = 25), 0.75% (n = 25)	30 mL (with epinephrine 1:400000)	None	a, b, c, h, i
Krone et al. (2001)	Canada	60	≥18	NR	elective arthroscopic shoulder surgery	Interscalene	Ropivacaine 0.125% (n = 20), 0.25% (n = 20), 0.5% (n = 20)	10 mL	GA	k, m, n
Zha et al. (2016)	China	99	18–80	I-II	elective arthroscopic shoulder surgery	Interscalene	Ropivacaine 0.75%, (n = 33); ropivacaine 0.5%, (n = 33); ropivacaine 0.25%, (n = 33)	6.7 mL	GA	a, b, e, g, k, o
								10 mL		
								20 mL		
Wang et al. (2022)	China	70	18–65	I-II	elective surgery of the forearm or hand	costoclavicular space	Ropivacaine 0.375% (n = 35), 0.5% (n = 35)	20 mL	GA	a, b, e, h, i, o
Venkatesh et al. (2016)	India	60	18–60	I-II	arm, forearm and hand surgery	supraclavicular	Ropivacaine 0.5% (n = 30), 0.75% (n = 30)	30 mL	None	a, b, h, i
Bertini et al. (1999)	Italy	60	18–60	I-III	elective surgery of the hand	axillary	Ropivacaine 0.5% (n = 30), 0.75% (n = 30)	32 mL	GA	a, b, e, g, h, i, n, o, p

a. Time to onset of sensory block.

b. Time to onset of motor block.

c. Time to first request for oral analgesia.

d. Degree of pain measured at the first requirement for postoperative analgesics.

e. Postoperative complications.

f. Pulmonary Function Changes.

g. Satisfaction.

h. Duration time of sensory blockade.

i. Duration time of motor blockade.

j. Cumulative opioid consumption.

k. Perioperative VAS, or NRPS, score.

l. Room air oxygen saturation.

m. Degree of sensory and motor blocks at different times.

n. Pain intensity and analgesic requirements in the hospital and at home.

o. Blocking success rate.

p. Need for intraoperative opioids.

### 3.3 Meta-analysis of primary outcomes

#### 3.3.1 Onset time of sensory blockade

**FIGURE 3:**
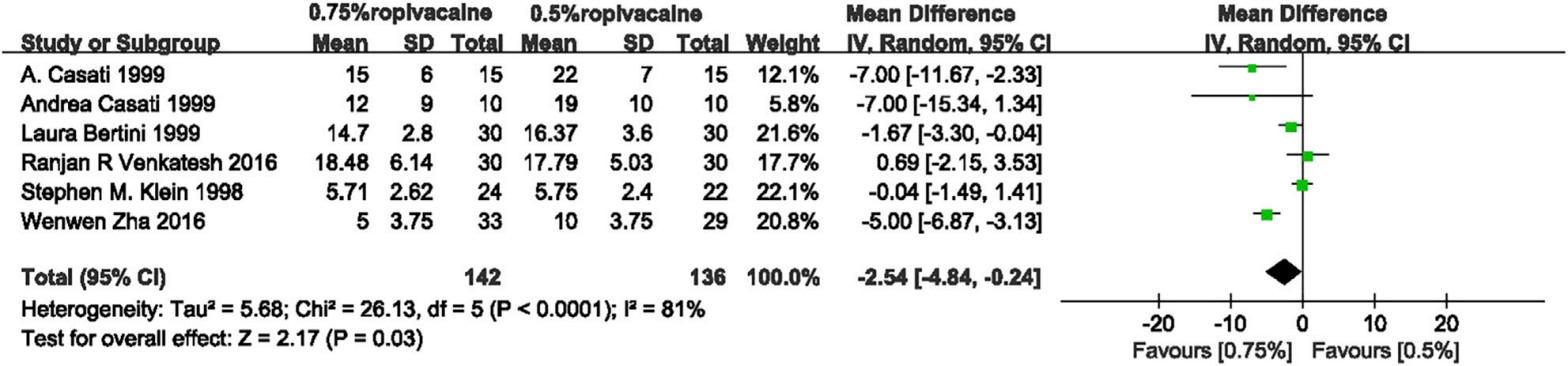
Onset time of sensory blockade comparing 0.75% ropivacaine and 0.5% ropivacaine. SD, standard deviation; IV, inverse variance method; CI, confidence interval.

The combined results of the six eligible studies (Klein et al., 1998; Casati et al., 1999a; Casati et al., 1999b; Bertini et al., 1999; Venkatesh et al., 2016; Wenwen et al., 2016) showed that, compared with 0.5% ropivacaine, 0.75% ropivacaine shortened the onset time of sensory blockade (weighted mean difference (WMD), −2.54; confidence interval (95% CI), −4.84 to −0.24; I^2^ = 81%; *p* < 0.0001; moderate quality of evidence) (Figure 3, Supplementary Material S3).

#### 3.3.2 Onset time of motor blockade

**FIGURE 4:**
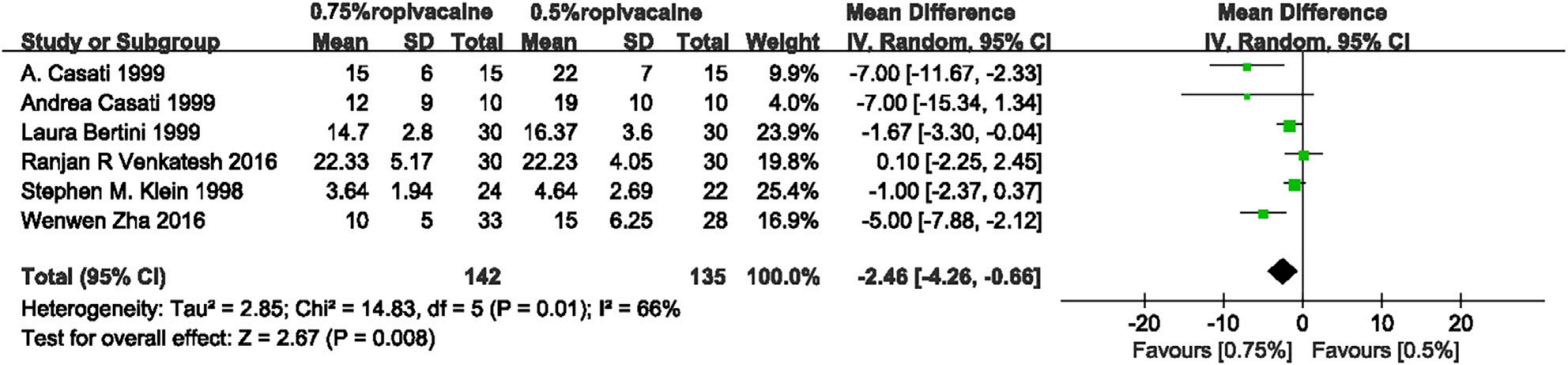
Onset time of motor blockade between 0.75% ropivacaine and 0.5% ropivacaine. SD, standard deviation; IV, inverse variance method; CI, confidence interval.

A comprehensive analysis of six studies (Klein et al., 1998; Casati et al., 1999a; Casati et al., 1999b; Bertini et al., 1999; Venkatesh et al., 2016; Wenwen et al., 2016) showed that 0.75% ropivacaine reduced the onset time of motor blockade when compared with 0.5% ropivacaine (WMD, −2.46; 95% CI, −4.26 to −0.66; I^2^ = 66%; *p* = 0.01; moderate quality of evidence) (Figure 4, Supplementary Material S3).

### 3.4 Meta-analysis of secondary outcomes

#### 3.4.1 Duration time of sensory blockade

**FIGURE 5:**
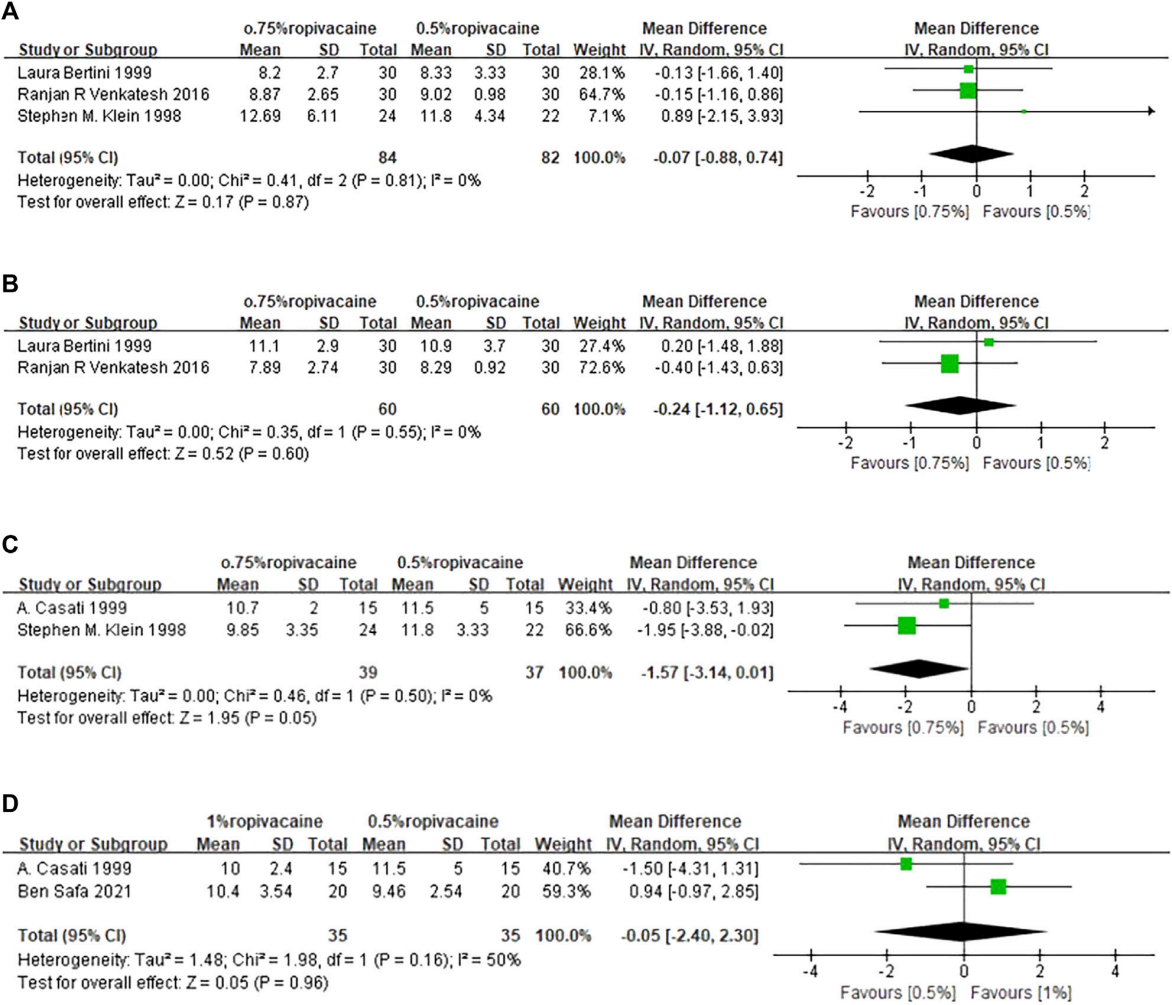
Meta-analysis results for secondary outcomes. **(A)**. Duration time of sensory blockade comparing 0.75% ropivacaine and 0.5% ropivacaine. **(B)**. Duration time of motor blockade comparing 0.75% ropivacaine vs. 0.5% ropivacaine. **(C)**. Time of first oral analgesia comparing 0.75% ropivacaine vs. 0.5% ropivacaine. **(D)**. Time of first oral analgesia comparing 0.5% ropivacaine vs. 1% ropivacaine. SD, standard deviation; IV, inverse variance method; CI, confidence interval.

The duration time of sensory blockade was reported in three included studies (Klein et al., 1998; Bertini et al., 1999; Venkatesh et al., 2016; Safa et al., 2021; Wang et al., 2023). Data from these studies (Klein et al., 1998; Bertini et al., 1999; Venkatesh et al., 2016) were pooled. Compared with 0.5% ropivacaine, the duration time of sensory blockade was similar to that of 0.75% ropivacaine (WMD, −0.07; 95% CI, −0.88 to 0.74; I^2^ = 0%; *p* = 0.81; high quality of evidence) (Figure 5A, Supplementary Material S3).

#### 3.4.2 Duration time of motor blockade

Data from two studies (Bertini et al., 1999; Venkatesh et al., 2016) showed no significant difference in the duration time of motor blockade between 0.75% ropivacaine and 0.5% ropivacaine (WMD, −0.24; 95% CI, −1.12 to 0.65; *p* = 0.55; high quality of evidence), with no significant evidence of heterogeneity (I^2^ = 0%, *p* = 0.55) (Figure 5B, Supplementary Material S3).

#### 3.4.3 Time to first request for oral analgesia (TFA)

Pooled results from 2 studies (Klein et al., 1998; Casati et al., 1999a) revealed no significant difference in the TFA between 0.5% and 0.75% ropivacaine (WMD, −1.57; 95% CI, −3.14 to 0.01; I^2^ = 0%; *p* = 0.5; moderate quality of evidence) (Figure 5C, Supplementary Material S3). The same was true for 0.5% ropivacaine and 1% ropivacaine (WMD, 0.17; 95% CI, −1.41 to 1.75; I^2^ = 50%; *p* = 0.16; very low quality of evidence) (Figure 5D, Supplementary Material S3).

## 4 Discussion

Taking the current evidence together, a few points can be summarized as follows: (1): In terms of the onset time of both sensory and motor blocks, moderate evidence showed that 0.75% ropivacaine was more effective than 0.5% ropivacaine. (2). With regard to the duration of both sensory and motor blockade, high evidence showed that 0.5% and 0.75% ropivacaine had similar effects (3). For the TFA, moderate to very low evidence showed that the effects of the three concentrations studied (0.5%, 0.75%, and 1% ropivacaine) were comparable.

Several studies also compared the effects of 0.5% ropivacaine with other concentrations other than 0.75%, but the data were not available. Wang et al. (Wang et al., 2023) compared the effects of 0.5% and 0.375% ropivacaine on the onset and duration time of sensory and motor blockade. Their results showed no significant difference in the onset time between these two concentrations (sensory: 15 [15–20] min *versus* 15 [13–20] min, *p* = 0.47; motor: 10 [10–15] min *versus* 10 [10–15] min, *p* = 0.61), but 0.5% ropivacaine produced a significantly longer duration of sensory (455 [398–490] min *versus* 610 [570–655] min, *p* < 0.001) and motor (470 [409–500] min *versus* 625 [578–665] min, *p* < 0.001) blockade than 0.375% ropivacaine. However, our pooled results showed that 0.75% and 0.5% ropivacaine provided similar durations of sensory and motor block, but 0.75% ropivacaine provided a shorter onset time of sensory and motor block. Interestingly, one study indicated that 0.5% and 1% ropivacaine provided comparable durations of sensory (13.8 ± 4.5 h *versus* 15.8 ± 6.3 h, *p >* 0.05) and motor blockade (14.9 ± 5.7 h *versus* 18.5 ± 9.7 h, *p >* 0.05) in another study (Safa et al., 2021). Combined with our results, it is suggested that 0.5%–1% ropivacaine may provide a similar sensory and motor block duration time but a longer block duration than 0.375% ropivacaine. Future studies are required to validate this. For TFA, a study (Krone et al., 2001) showed that the TFAs were similar among three concentrations of ropivacaine of 0.125%, 0.25%, and 0.5% (674 ± 55 min *versus* 613 ± 241 min *versus* 649 ± 248 min, *p >* 0.05). Similarly, our pooled results showed no difference in TFA between the 0.5% and 0.75% ropivacaine groups. With regard to side effects, two studies (Safa et al., 2021; Wang et al., 2023) demonstrated no difference in postoperative nausea and vomiting between the 0.5% vs 0.375% (0% vs 9%, *p >* 0.05) and 0.5% vs 1% (33.33% vs 46.67%, *p* = 0.83) ropivacaine groups, respectively. One study (Wenwen et al., 2016) reported sleep quality, and the results showed no significant difference among the 0.25%, 0.5%, and 0.75% ropivacaine groups (sleep disturbance because of pain, 15% *versus* 7% *versus* 18%, *p* = 0.416). Two studies (Bertini et al., 1999; Wenwen et al., 2016) reported that the block satisfaction in 0.25%, 0.5% and 0.75% ropivacaine was similar. Collectively, these findings suggest that increasing concentrations of 0.75% ropivacaine may not increase the risks of side effects. These evidences support the safety of 0.75% ropivacaine use for BPB. However, this conclusion should be confirmed due to the relatively small number of studies. In a word, above studies provided more reference and research basis for the selection of ropivacaine concentration for clinic in adult brachial plexus block, which is conducive to further exploring the optimal concentration of ropivacaine for brachial plexus block in the future.

Several studies have compared the effects of ropivacaine with other local anesthetics for BPB in upper limb surgeries. Compared with bupivacaine, ropivacaine at lower concentrations (0.5%) has a higher degree of separation between motor and sensory blockade (Markham et al., 1996). McClellan et al. (McClellan and Faulds, 2000) showed that 30–40 mL of 0.5% ropivacaine produced brachial plexus anesthesia similar to that produced by an equivalent volume of 0.5% bupivacaine in patients who underwent upper limb surgeries. However, the onset of sensory block with ropivacaine tended to be faster, and the duration of the motor block was shorter (McClellan and Faulds, 2000). Singelyn et al. (Singelyn, 2001) reported that the minimum effective concentration of ropivacaine is 0.5%; however, the benefits of increasing the concentration to 0.75% or 1% remain controversial. Our study conducted a synthetic and quantitative analysis of the evidence on the effects of different concentrations of ropivacaine in BPB in upper limb surgeries, indicating that 0.75% ropivacaine is a preferred concentration to 0.5% ropivacaine for BPB in upper limb surgeries because of the shortened onset time of motor and sensory blockade.

In clinical practice, BPB can be achieved through several routes, including interscalene, costoclavicular space, supraclavicular, and axillary approaches (Bhat et al., 2020; Jones et al., 2020; Mojica et al., 2022). The diffusion rate of local anesthetics, the speed of vascular absorption, and the blocking sequence are different for different approaches, which will influence the effects of brachial plexus block (Wenwen et al., 2016; Safa et al., 2021). Studies have found that different approaches may produce different blocking effects even using the same concentration and dose of ropivacaine (Bhat et al., 2020; Jones et al., 2020). Additionally, the volume of ropivacaine used for brachial plexus blocks also varies. The volume of local anesthetics can affect the efficiency and duration of blockage (Wenwen et al., 2016; Safa et al., 2021). Therefore, the effect of the block may vary between different volumes of ropivacaine with the same concentration or same approach (Chen et al., 2021; Gao et al., 2022). It is thus worthwhile to examine the optimal concentration of ropivacaine for different BPB approaches and/or injection volumes. In this meta-analysis, interscalene BPB was performed in 4 RCTs (Klein et al., 1998; Casati et al., 1999a; Casati et al., 1999b; Wenwen et al., 2016), and supraclavicular (Venkatesh et al., 2016) and axillary (Bertini et al., 1999) approaches were performed in one RCT. For volume injection, 10 mL ropivacaine was used in 1 RCT (Wenwen et al., 2016), 20 mL ropivacaine in 2 RCTs (Casati et al., 1999a; Casati et al., 1999b), and 30 mL ropivacaine in 3 RCTs (Klein et al., 1998; Bertini et al., 1999; Venkatesh et al., 2016). We conducted subgroup analysis based on the BPB approaches and ropivacaine volumes, and the results showed that different approaches or ropivacaine volumes did not affect the comparison results between 0.75% and 0.5% ropivacaine (Supplementary Material S4). However, due to the limited number of included studies, these findings need to be further verified. Therefore, more well-designed RCTs were acquired to test the optimal ropivacaine concentration or volumes for different BPB approaches.

We chose 0.5% ropivacaine as the control group because it was the most studied concentration for BPB. Six studies regarding the clinical effectiveness of different ropivacaine concentrations for BPB in upper limb surgeries were excluded because they did not explore the established control concentration of ropivacaine (Krenn et al., 2003; Fredrickson et al., 2012; Nishiyama, 2012; Fang et al., 2016; Wong et al., 2016; Bhat et al., 2020). Fredrickson et al. (Fredrickson et al., 2012) reported that 0.75% ropivacaine was superior to 0.375% ropivacaine in terms of block duration, but no significant difference was found between these two concentrations in terms of analgesic consumption, postoperative NRPS scores, incidence of postoperative dyspnea, and block satisfaction (Fredrickson et al., 2012). Nishiyama et al. (Nishiyama, 2012) reported that 0.375% and 0.75% ropivacaine generated similar onsets and durations of sensory and motor blocks. However, it should be noted that the authors acknowledged that one important limitation in this study (Nishiyama, 2012) was that motor and sensory blocks were examined roughly, which may influence the results. Nevertheless, more well-designed studies are needed to confirm these conclusions. Bhat et al. (Bhat et al., 2020) concluded that there was no significant difference between 0.1285% and 0.15% ropivacaine in terms of the onset of sensory block and complication rate; however, 0.15% ropivacaine was superior regarding the analgesic requirements and block satisfaction. In a study by Krenn et al. (Krenn et al., 2003), although 0.3% ropivacaine shortened the onset time of motor blockade compared with 0.25% and 0.1875% ropivacaine, no significant difference was found in the onset time of sensory blockade and satisfaction.

Several adjuvants, such as adrenaline, dexamethasone, and dexmedetomidine, are commonly added to ropivacaine for BPB to achieve better analgesic effects (Bharti et al., 2015; Kumari et al., 2020; Grelet et al., 2021; Lee et al., 2021; Mar et al., 2021; Pande et al., 2021; Venkatraman et al., 2021; Xuan et al., 2021). Therefore, we further explored the possible effects of adding adjuvants to ropivacaine on our results. Among the included RCTs, data from Klein et al. (Klein et al., 1998) most likely affected the pooled outcome due to the use of adrenaline in ropivacaine and therefore may influence the results of the onset time of motor and sensory blockade. However, sensitivity analysis revealed that it had no obvious effects on the results. As adjuvants were only used in one included study, we did not conclude whether adding adjuvants to local anesthetics influenced the comparative results between different concentrations of ropivacaine. Therefore, the exact effects of adjuvants in combination with different concentrations of ropivacaine need to be determined in future studies.

This study had some limitations. First, the number of included studies and the sample size were relatively small. Second, the heterogeneity was relatively high for several indicators, but their resources were not well determined. Finally, the vast majority of data were pooled for meta-analysis between the 0.5% and 0.75% ropivacaine groups, and comparisons with other concentrations of ropivacaine were not available to be explored. Therefore, large-sample, multicenter, well-designed RCTs are required to investigate the optimal concentration of ropivacaine for BPB, which will provide longer analgesia with a safer profile.

## 5 Conclusion

In summary, moderate-quality evidence suggested that 0.75% ropivacaine is a better choice for brachial nerve blockade in adult patients during upper limb surgeries because it provides a faster onset of sensory and motor blockade.

## Data Availability

The original contributions presented in the study are included in the article/Supplementary Material, further inquiries can be directed to the corresponding authors.

## Abbreviations

ASA, American Society of Anesthesiologists; BPB, Brachial Plexus Block; NR, Not Reported; GA, General Anesthesia; VAS, Visual Analogue Scale; NRPS, The Numerical Rating Pain Scale.
