# Supplementary material for: Optimal concentration of ropivacaine for brachial plexus blocks in adult patients undergoing upper limb surgeries: a systematic review and meta-analysis
Source: Front Pharmacol. 2023 Nov 16;14:1288697. doi: 10.3389/fphar.2023.1288697 (PMC10687368; doi:10.3389/fphar.2023.1288697)
Supplement: Supplementary file 2 [file Table1.DOCX]

**Scope statement**

*Frontiers in pharmacology* is the leading journal in the field of pharmacology, disseminating cutting-edge scientific knowledge and influential discoveries on the interactions between chemicals and organisms. The purpose of this study was to determine the optimal concentration of ropivacaine in brachial plexus blocks in adult patients undergoing upper extremity surgeries, which will provide evidence for the concentration selection of ropivacaine in brachial plexus blocks. Based on the above considerations, we believe that the current work is well suited for publication in *Frontiers in Pharmacology* and that the novelty and quality of this study reached the high standards of *Frontiers in Pharmacology*. Our contribution will appeal to a wide readership of your journal.
