# Supplementary material for: Optimal concentration of ropivacaine for brachial plexus blocks in adult patients undergoing upper limb surgeries: a systematic review and meta-analysis
Source: Front Pharmacol. 2023 Nov 16;14:1288697. doi: 10.3389/fphar.2023.1288697 (PMC10687368; doi:10.3389/fphar.2023.1288697)
Supplement: Supplementary file 4 [file Table7.DOCX]

Table 1. Characteristics of included studies

| Study | country | Sample | Age (yr) | ASA | Surgical procedure | BPB approach | BPB | Injection volume | Anesthesia | Outcomes |
| --- | --- | --- | --- | --- | --- | --- | --- | --- | --- | --- |
| Casati et al.1999 | Italy | 45 | 18-65 | I-II | elective shoulder surgery | Interscalene | Ropivacaine 0.5%(n=15),0.75%(n=15),1%(n=15) | 20 ml | NR | a, b, c, d, e; |
| Casati et al.1999 | Italy | 20 | 18-65 | I-II | elective shoulder  surgery | Interscalene | Ropivacaine  0.5%(n=10),0.75%(n=10) | 20 ml | None | a, b, d, e, f, g; |
| Safa et al.2021 | Canada | 40 | 18-80 | I-III | arthroscopic shoulder surgery | Interscalene | Ropivacaine  0.5%(n=20),1%(n=20) | 5 ml | GA | c, e, h, i, j, k, l; |
| Klein et al.1998 | North Carolina(USA) | 50 | ≥18 | I-III | outpatient shoulder surgery | Interscalene | Ropivacaine  0.5%(n=25),0.75%(n=25) | 30ml (with epinephrine 1:400000) | None | a, b, c, h, i; |
| Krone et al.2001 | Canada | 60 | ≥18 | NR | elective arthroscopic shoulder surgery | Interscalene | Ropivacaine  0.125%(n=20),0.25%(n=20),0.5%(n=20) | 10ml | GA | k, m, n; |
| Zha et al.2016 | China | 99 | 18-80 | I-II | elective arthroscopic shoulder surgery | Interscalene | Ropivacaine 0.75%, (n=33); ropivacaine 0.5%, (n=33); ropivacaine 0.25%, (n=33) | 6.7ml; 10ml;  20ml | GA | a, b, e, g, k, o; |
| Wang et al.2022 | China | 70 | 18-65 | I-II | elective surgery of the forearm or hand | costoclavicular space | Ropivacaine  0.375%(n=35),0.5%(n=35) | 20ml | GA | a, b, e, h, i, o; |
| Venkatesh et al.2016 | India | 60 | 18-60 | I-II | arm, forearm  and hand surgery | supraclavicular | Ropivacaine  0.5%(n=30),0.75%(n=30) | 30ml | None | a, b, h, i; |
| Bertini et al.1999 | Italy | 60 | 18-60 | I-III | elective surgery of the hand | axillary | Ropivacaine  0.5%(n=30),0.75%(n=30) | 32ml | GA | a, b, e, g, h, i, n, o, p; |

1. Time to onset of sensory block.
2. Time to onset of motor block.
3. Time to first request for oral analgesia.
4. Degree of pain measured at the first requirement for postoperative analgesic.
5. Postoperative complications
6. Pulmonary Function Changes
7. Satisfaction
8. Duration time of sensory blockade
9. Duration time of motor blockade
10. Cumulative opioid consumption
11. Perioperative VAS or NRPS score
12. Room air oxygen saturation
13. Degree of sensory and motor blocks in different times
14. Pain intensity and analgesic requirement in hospital and at home
15. Block successful rate
16. Need for intraoperative opioids

Abbreviations: ASA, American Society of Anesthesiologist; BPB, Brachial Plexus Block; NR, Not Reported; GA, General Anesthesia; VAS, Visual Analogue Scale; NRPS, The Numerical Rating Pain Scale.
