# Supplementary material for: Optimal concentration of ropivacaine for brachial plexus blocks in adult patients undergoing upper limb surgeries: a systematic review and meta-analysis
Source: Front Pharmacol. 2023 Nov 16;14:1288697. doi: 10.3389/fphar.2023.1288697 (PMC10687368; doi:10.3389/fphar.2023.1288697)
Supplement: Supplementary file 5 [file Table2.DOCX]

**Search strategies**

**PUBMED**

1.((Interscalene[Title/Abstract]) OR (costoclavicular space[Title/Abstract]) OR (Supraclavicular[Title/Abstract]) OR (axillary[Title/Abstract]) OR (Brachial Plexus Block[MeSH Terms]) OR (Brachial Plexus Block*[Title/Abstract]) OR (nerve block[MeSH Terms]) OR (nerve block[Title/Abstract]) OR (peripheral nerve blockade[Title/Abstract]) OR (brachial plexus anaesthesia[Title/Abstract]) OR (Brachial Plexus Block anesthesia[Title/Abstract]) OR (regional*[Title/Abstract]) OR (local*[Title/Abstract]) OR (nerv* block*[Title/Abstract]))

2. ((Ropivacaine[MeSH Terms]) OR (Ropivacaine[Title/Abstract]) OR (ropivacain*[Title/Abstract]))

3. (concentration[Title/Abstract])

4. (((randomized-controlled-trial[Title/Abstract]) OR (randomization[Title/Abstract]) OR (controlled-study[Title/Abstract]) OR (multicenter-study[Title/Abstract]) OR (phase-3-clinical-trial[Title/Abstract]) OR (phase-4-clinical-trial[Title/Abstract]) OR (double blind-procedure[Title/Abstract]) OR (single-blind-procedure[Title/Abstract]) OR (random*[Title/Abstract]) OR (cross*over*[Title/Abstract]) OR (placebo*[Title/Abstract]) OR (volunteer*[Title/Abstract]) OR (controlled clinical trial[Title/Abstract]) OR (blind*[Title/Abstract])) NOT (animals[Title/Abstract]))

5. #1 AND #2 AND #3 AND #4

**Cochrane library Trials**

#1 MeSH descriptor: [Brachial Plexus Block| explode all trees

#2 (Brachial Plexus Block*):ti,ab,kw

#3 (peripheral nerve blockade):ti,ab,kw

#4 (brachial plexus anaesthesia):ti,ab,kw

#5 (Brachial Plexus Block anesthesia):ti,ab,kw

#6 (Interscalene):ti,ab,kw

#7 (costoclavicular space):ti,ab,kw

#8 (Supraclavicular):ti,ab,kw

#9 (axillary):ti,ab,kw

#10 #1 OR#2 OR#3 OR#4 OR#5 OR#6 OR #7 OR #8 OR#9

#11 MeSH descriptor. [Ropivacaine] explode al trees

#12 (ropivacaine):ti,ab,kw

#13 (ropivacaine*):ti,ab,kw

#14 #11 OR#12 OR#13

#15 (concentration):ti,ab,kw

#16 (concentrat*):ti,ab,kw

#17 #15 OR#16

#18 (randomized-controlled-trial):ti,ab,kw

#19 (randomization):ti,ab,kw

#20 (contolled-study):ti,ab,kw

#21 (mulicenter-study):ti,ab,kw

#22 (single-blind-procedure):ti,ab,kw

#23 (double blind-procedure):ti,ab,kw

#24 (random*):ti,ab,kw

#25 (volunteer*):ti,ab,kw

#26 (controlled clinical trial):ti,ab,kw

#27 (blind*):ti,ab,kw

#28 (animals):ti,ab,kw

#29 (#18 OR#19 OR #20 OR#21 OR#22 OR#23 0R #24 OR25 OR #26 OR#27) NOT#28

#30 #10 AND#14 AND#17 AND #29

**EMBASE**

#1 ‘brachial plexus anesthesia’/exp OR ’brachial plexus anesthesia’:ti,ab,kw OR’ brachial block anesthesia’:ti,ab,kw OR ’brachial plexus block anesthesia’:ti,ab,kw OR’ peripheral neve block’:ti,ab,kw OR’ brachial plexus blockade’:ti,ab,kw OR Interscalene

:ti,ab,kw OR ‘costoclavicular space’:ti,ab,kw OR supraclavicular:ti,ab,kw OR axillary

:ti,ab,kw

#2 ‘ropivacaine’/exp OR ropivacaine’:ti,ab,kw OR ropivacain*:ti,ab,kw

#3 ‘concentration’/exp OR concentration:ti,ab,kw OR concentrat*:ti,ab,kw

#4 ‘randomized controlled trial’/exp OR randomized:ti,ab,kw OR ‘randomized controlled trial’:ti,ab,kw OR ‘trial’,randomized controlled’:ti,ab,kw OR randomization:ti,ab,kw OR ‘controlled study’:ti,ab,kw OR ‘mulicenter study’:ti,ab,kw OR ‘double blind procedure’:ti,ab,kw OR ‘single blind procedure’:ti,ab,kw OR placebo:ti,ab,kw OR volunteer*:ti,ab,kw OR ‘controlled clinical trial’:ti,ab,kw OR blind*:ti,ab,kw

#5 #1 AND #2 AND #3 AND #4

**Web of science**

#1 Brachial Plexus Block(Topic) or Brachial Plexus Block*(Topic) or nerve block(Topic) or peripheral nerve blockade(Topic) or brachial plexus anaesthesia(Topic) or Brachial Plexus Block anesthesia(Topic) or nerv* block*(Topic) or Interscalene(Topic) or costoclavicular space(Topic) or Supraclavicular(Topic) or axillary(Topic)

#2 (TS=(randomized-controlled-trial) OR TS=(randomization) OR TS=(controlled-study) OR TS=(multicenter-study) OR TS=(phase-3-clinical-trial) OR TS=(phase-4-clinical-trial) OR TS=(double blind-procedure) OR TS=(single-blind-procedure) OR TS=(random*) OR TS=(placebo*) OR TS=(volunteer)OR TS=(controlled clinical trial) OR TS=(blind)) NOT TS=(animals)

#3 concentration(Topic) or concentrat*(Topic)

#4 ropivacaine(Topic) or ropivacain*(Topic)

#5 #4 AND #3 AND #2 AND #1
