# Supplementary material for: Optimal concentration of ropivacaine for brachial plexus blocks in adult patients undergoing upper limb surgeries: a systematic review and meta-analysis
Source: Front Pharmacol. 2023 Nov 16;14:1288697. doi: 10.3389/fphar.2023.1288697 (PMC10687368; doi:10.3389/fphar.2023.1288697)
Supplement: Supplementary file 7 [file Table4.DOCX]

| **Results for brachial plexus block in adult patients undergoing upper limb surgery: a systematic review and meta-analysis** | | | | | | |
| --- | --- | --- | --- | --- | --- | --- |
| **Patient or population:** patients with brachial plexus block in adult patients undergoing upper limb surgery: a systematic review and meta-analysis **Settings:**  **Intervention:** Results | | | | | | |
| **Outcomes** | **Illustrative comparative risks* (95% CI)** | | **Relative effect (95% CI)** | **No of Participants (studies)** | **Quality of the evidence (GRADE)** | **Comments** |
|  | Assumed risk | Corresponding risk |  |  |  |  |
|  | **Control** | **Results** |  |  |  |  |
| **Onset time of motor blockade(min)** |  | The mean onset time of motor blockade(min) in the intervention groups was **2.46 lower** (4.26 to 0.66 lower) |  | 277 (6 studies) | ⊕⊕⊕⊝ **moderate**^1^ |  |
| **Onset time of sensory blockade(min)** |  | The mean onset time of sensory blockade(min) in the intervention groups was **2.54 lower** (4.84 to 0.24 lower) |  | 278 (6 studies) | ⊕⊕⊕⊝ **moderate**^1^ |  |
| **Duration time of sensory blockade(hours)** |  | The mean duration time of sensory blockade(hours) in the intervention groups was **0.07 lower** (0.88 lower to 0.74 higher) |  | 166 (3 studies) | ⊕⊕⊕⊕ **high** |  |
| **Duration time of motor blockade(hours)** |  | The mean duration time of motor blockade(hours) in the intervention groups was **0.24 lower** (1.12 lower to 0.65 higher) |  | 120 (2 studies) | ⊕⊕⊕⊕ **high** |  |
| **Time of first oral analgesia(h)0.5% vs 0.75%** |  | The mean time of first oral analgesia(h)0.5% vs 0.75% in the intervention groups was **1.57 lower** (3.14 lower to 0.01 higher) |  | 76 (2 studies) | ⊕⊕⊕⊝ **moderate**^2^ |  |
| **Time of first oral analgesia(h)0.5% vs 1%** |  | The mean time of first oral analgesia(h)0.5% vs 1% in the intervention groups was **0.17 higher** (1.41 lower to 1.75 higher) |  | 70 (2 studies) | ⊕⊝⊝⊝ **very low**^1,2,3^ |  |
| *The basis for the **assumed risk** (e.g. the median control group risk across studies) is provided in footnotes. The **corresponding risk** (and its 95% confidence interval) is based on the assumed risk in the comparison group and the **relative effect** of the intervention (and its 95% CI).  **CI:** Confidence interval; | | | | | | |
| GRADE Working Group grades of evidence **High quality:** Further research is very unlikely to change our confidence in the estimate of effect.  **Moderate quality:** Further research is likely to have an important impact on our confidence in the estimate of effect and may change the estimate. **Low quality:** Further research is very likely to have an important impact on our confidence in the estimate of effect and is likely to change the estimate. **Very low quality:** We are very uncertain about the estimate. | | | | | | |
| ^1^ Large I^2^-value ^2^ Small sample size ^3^ High risk of selective reporting bias | | | | | | |
