# Supplementary material for: Optimal concentration of ropivacaine for brachial plexus blocks in adult patients undergoing upper limb surgeries: a systematic review and meta-analysis
Source: Front Pharmacol. 2023 Nov 16;14:1288697. doi: 10.3389/fphar.2023.1288697 (PMC10687368; doi:10.3389/fphar.2023.1288697)
Supplement: Supplementary file 8 [file Table6.DOCX]

Supplementary Material 4. Forest plots


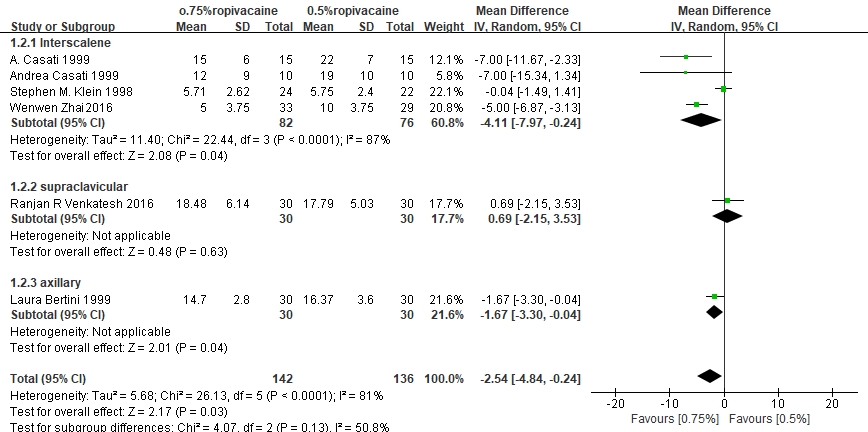
Figure.1 Subgroup analysis regarding onset time of sensory blockade according to different brachial plexus block approaches between 0.75% ropivacaine and 0.5% ropivacaine. SD, standard deviation; IV, inverse variance method; CI, confidence interval.


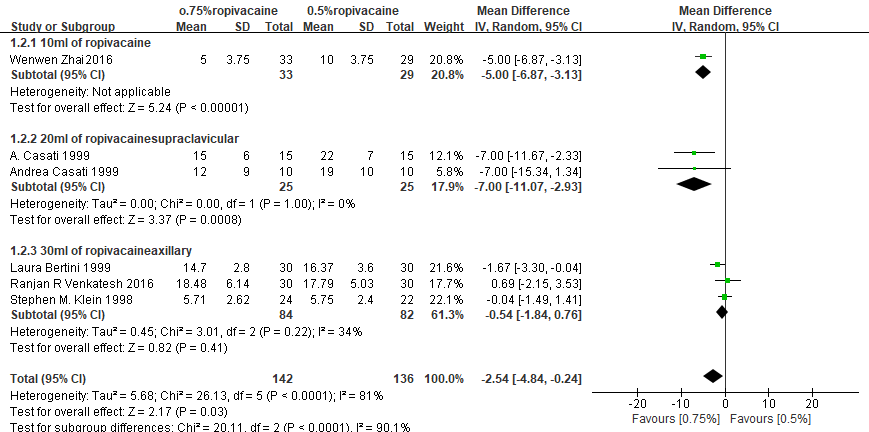


Figure.2 Subgroup analysis of onset time of sensory blockade according to different injection volume of ropivacaine. SD, standard deviation; IV, inverse variance method; CI, confidence interval.


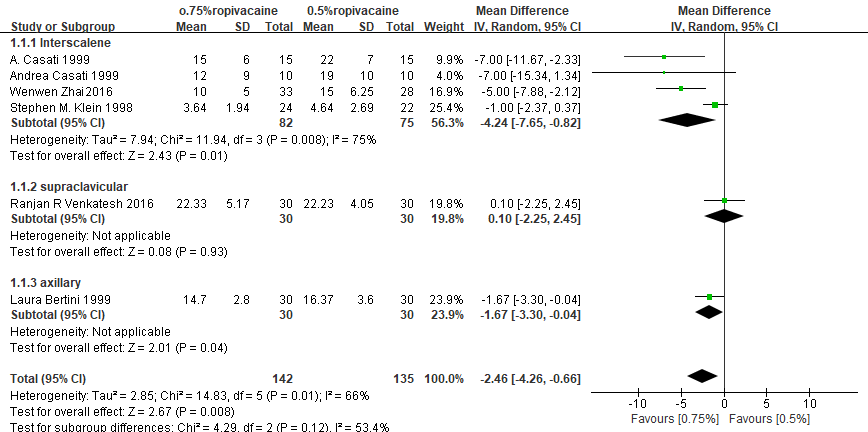


Figure.3 Subgroup analysis regarding onset time of motor blockade according to different brachial plexus block approaches between 0.75% ropivacaine and 0.5% ropivacaine. SD, standard deviation; IV, inverse variance method; CI, confidence interval.


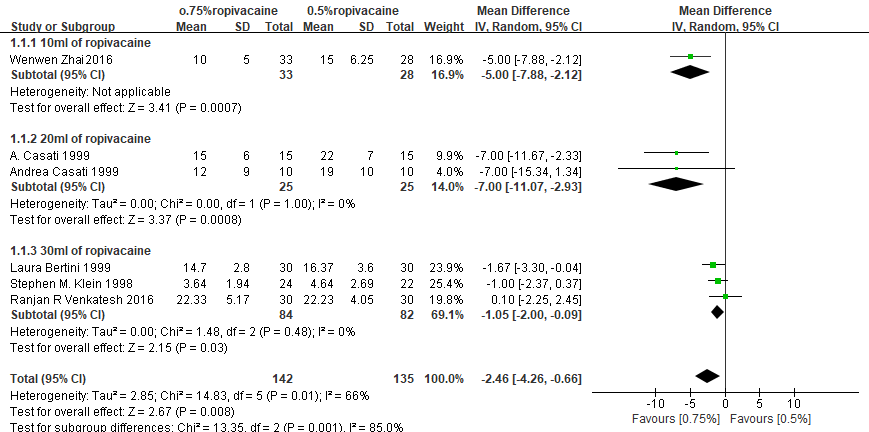


Figure.6 Subgroup analysis of onset time of motor blockade according to different injection volume of ropivacaine. SD, standard deviation; IV, inverse variance method; CI, confidence interval.
