# Supplementary material for: Optimal concentration of ropivacaine for brachial plexus blocks in adult patients undergoing upper limb surgeries: a systematic review and meta-analysis
Source: Front Pharmacol. 2023 Nov 16;14:1288697. doi: 10.3389/fphar.2023.1288697 (PMC10687368; doi:10.3389/fphar.2023.1288697)
Supplement: Supplementary file 3 [file DataSheet1.ZIP › 信息提取表(模板).doc]

**附件二：**

**基本信息**

**Date（提取日期）	Reviewer（提取者姓名）:**

**Study Title（文献标题）**

| First author**第一作者** |  |
| --- | --- |
| Year of publication **出版年** |  |
| Country of publication **出版国家** |  |
| Publication type**出版的类型** | Journal |
| **作者联系方式**  （一些文献能够获取的信息不全，需要联系作者） |  |

**纳入文**献的合格性

| **研究是否为随机对照** | 是： 不是： |
| --- | --- |
| **参与者是否为诊断明确的患者** |  |
| **干预措施是否符合要求** | 是：___；不是：_______ |
| **备注：____________________________________________________________________________**  （如果研究不是随机对照干预文献即排除，注明排除的理由就好） | |

**研究对象的特征（参与者的总人数及各组人 数、人口学信息（性别、年龄等）、研究地点、纳入 人群的基础状况、有无合并症，以及纳入排除标准 等**）

| 研究对象的特征 | | Study |
| --- | --- | --- |
| **参与者的总人数及干预和对照组的各组人 数** | |  |
| **研究对象** | | - ？入组，。。。。。的患者 - 各组对象基线一致？（p＞0.05）   纳入对象基线表（如下表）   - Other参与者的其他基本情况：比如患糖尿病的老年人 - 有无合并症： |
| **研究对象纳入标准** | |  |
| **研究对象排除标准** | |  |
| **参与者的国家/研究地点** | | 如来自美国的研究，在 社区照顾中心或家庭中生活的老年人 |
| **干预组的措施**  (包括持续的时间) | | 记录干预措施的具体内容、方式、操 作规范，参与人员、干预 的时间、频率、周期，参与干预的人员是否接受培 训等信息。 |
| **对照组的措施**  (包括持续的时间) | | 记录对照组措施的具体内容、方式、操 作规范，参与人员、干预 的时间、频率、周期，参与干预的人员是否接受培 训等信息。 |
| **随访时间** | | 如随访3个月、6个月 |
| **结果数据** | **测量结果** | 见后面图表 |

## 方法Methods: Trial Quality：随机方案的产生，分配方案的隐 藏，是否使用盲法，是否存在结果数据的不完整，是 否选择性报告结果，及是否存在其它偏倚

| **随机对照数字的产生**  （对于分配序列的生成尽可能详细地说明分配序列的生成方法） | **RCT**  **-低风险**：计算机随机数字生成器 ，扔硬币，洗牌的卡片和信封，掷骰子，抽签等  **-高风险**：通过奇偶或出生日期产生序列 ，通过入院日期产生序列等  **- Unclear** |
| --- | --- |
| Allocation concealment  **分配方案的隐藏**  （分配隐藏尽可能详细地描述隐藏随机分配序列的方法，并陈述隐藏 分配序列的可信度） | **·Yes** 低风险 第三方中心分配，顺序编号、不透明、密封的信封等  ·**Unclear**  **·No** 高风险 使用摊开的随机分配表（如随机序列清单），交替或循环，出生日期等 |
| Blinding  **是否使用盲法**  因参与者和实施者了解干预情况而导致实施偏倚 | - Patients Yes No Not stated - Manipulator Yes No Not stated - Outcomes collectors Yes No Not stated - Data analysts Yes No Not stated |
| Outcome assessment and measurement  **结果数据的完整性** | **·Yes低风险**：无缺失数据；缺失数据的数目在各干预组相当且各组缺失原因类似；  对二分类变量，与观察事件的发生风险相比，缺失比例不足以影响预估的干预效应；  对连续性结局数据，缺失数据的合理效应规模（均数差或标准均数差）不会大到影响观察的效应规模;  **·No** **高风险**：缺失数据的产生很大可能与真实结局相关, 缺失数据的数目及缺失原因在各干预组相差较大  对二分类变量，与观察事件的发生风险相比，缺失比例足以影响预估的干预效应  对连续性结局数据，缺失数据的合理效应规模（均数差或标准均数差）足以影响观察的效应规模;  ·**Unclear** |
| **是否选择性报告结果** | ·**Yes高风险**不是所有的预先说明的首要结局均被报道 ；一个或多个首要结局为采用预先说明的测量方法、分析方法或数据子集来报道；系统评价感兴趣的一个或多个首要结局报道不全，以至于不能纳入meta分析；  ·**No 低风险**发表的报告包括所有的结局，包括预先说明的结局；  ·**Unsure** |
| **是否存在其它偏倚** | 具有与特殊试验设计相关的潜在偏倚来源，或被指欺诈， 或其它问题 Percent ____ |
| Notes  **备注** | Compliance  **依从性等** |

## 注意： 对方法学资料除“Yes”、“No”、“Unclear”选项外，还应 提示评价者尽可能引用原文所提供的语句，要尽可能对一些重要细 节进行详细描述。

## Results结果

Comparison:	一是进行的是什 么样的比较，二是每一比较中使用的结局指标有哪些，三是结果资料的类型。

Outcome（结果数据）:

Subcategory（亚组）:

主要结局指标、次要结局指标

|  | **干预组:** | **对照组:** |
| --- | --- | --- |
| **随机总数** |  |  |
| **排除数** |  |  |
| **观察数** |  |  |
| **失访数** |  |  |
| **发生事件数** |  |  |

Note：

| **二分类变量**组别 | 发生事件数 | 未发生事件数 | 总计 |
| --- | --- | --- | --- |
| 干预 |  |  |  |
| 对照组 |  |  |  |

| **连续性变量（干预前）** | 干预组 | | | 对照组 | | |
| --- | --- | --- | --- | --- | --- | --- |
| Outcomes | N | Mean | SD | N | Mean | SD |
| 举例：抑郁情绪 |  |  |  |  |  |  |
|  |  |  |  |  |  |  |
|  |  |  |  |  |  |  |
|  |  |  |  |  |  |  |
| **连续性变量（干预后）** | 干预组 | | | 对照组 | | |
| Outcomes | N | Mean | SD | N | Mean | SD |
| 举例：抑郁情绪 |  |  |  |  |  |  |
|  |  |  |  |  |  |  |
|  |  |  |  |  |  |  |
|  |  |  |  |  |  |  |
| **连续性变量（改变值）** | 干预组 | | | 对照组 | | |
| Outcomes | N | Mean | SD | N | Mean | SD |
| 举例：抑郁情绪 |  |  |  |  |  |  |
|  |  |  |  |  |  |  |
|  |  |  |  |  |  |  |
|  |  |  |  |  |  |  |

Note：

提取混杂因素：

基金来源?

作者得出的关键性结论?

作者对混杂因素的评价等?
