# Supplementary material for: Optimal concentration of ropivacaine for brachial plexus blocks in adult patients undergoing upper limb surgeries: a systematic review and meta-analysis
Source: Front Pharmacol. 2023 Nov 16;14:1288697. doi: 10.3389/fphar.2023.1288697 (PMC10687368; doi:10.3389/fphar.2023.1288697)
Supplement: Supplementary file 3 [file DataSheet1.ZIP › 肋锁间隙入路 1/0.5%罗哌/Comparison of the efficacy of costoclavicular space brachial plexus blockade with 0.5% versus 0.375% ropivacaine a randomized, double-blind, single-centre, noninferiority clinical trial.doc]

**附件二：**

**基本信息**

**Date（提取日期）	Reviewer（提取者姓名）: 2023 01.10 吴琳**

**Study Title（文献标题）Comparison of the efﬁcacy of costoclavicular space brachial plexus blockade with 0.5% versus 0.375% ropivacaine: a randomized,**

**double-blind, single-centre, noninferiority clinical trial**

| First author**第一作者** | Shuang Wang |
| --- | --- |
| Year of publication **出版年** | 2022 |
| Country of publication **出版国家** | China |
| Publication type**出版的类型** | Journal |
| **作者联系方式**  （一些文献能够获取的信息不全，需要联系作者） | linchunshui2014@163.com |

**纳入文**献的合格性

| **研究是否为随机对照** | 是：✔ 不是： |
| --- | --- |
| **参与者是否为诊断明确的患者** | ✔ |
| **干预措施是否符合要求** | 是：_✔__；不是：_______ |
| **备注：____________________________________________________________________________**  （如果研究不是随机对照干预文献即排除，注明排除的理由就好） | |

**研究对象的特征（参与者的总人数及各组人 数、人口学信息（性别、年龄等）、研究地点、纳入 人群的基础状况、有无合并症，以及纳入排除标准 等**）

| 研究对象的特征 | | Study |
| --- | --- | --- |
| **参与者的总人数及干预和对照组的各组人 数** | | 70人。拟在CCS BPB下行前臂或手部手术  （1）20ml 0.375% Ropivacaine  （2）20ml 0.5% Ropivacaine   |
| **研究对象** | | - 2020.5.9-2020-9.30入组，拟在CCS BPB下行前臂或手部手术的患者。 - 各组对象基线一致（p＞0.05）   纳入对象基线表（如下表）   |
| **研究对象纳入标准** | | 1、 年龄 18-65岁 2、 ASA I-II级 3、 拟在CCS BPB下行前臂或手部手术 4、 自愿参加 5、 知情同意 |
| **研究对象排除标准** | | 1、 有局麻药过敏史 2、 神经损伤或患肢感觉异常 3、 锁骨下窝手术史 4、 有出血倾向或凝血障碍 5、 针刺部位的损伤或感染 6、 精神、语言或听力障碍 7、 近三个月参与了其他临床试验 |
| **参与者的国家/研究地点** | | 来自中国的研究，拟在CCS BPB下行前臂或手部手术的患者 |
| **干预组的措施**  (包括持续的时间) | | （1）20ml/组/人。阻滞后30mins 感觉+运动阻滞评分＜16分则认为阻滞失败，数据剔除。 |
| **对照组的措施**  (包括持续的时间) | |  |
| **随访时间** | | 如果出院时仍有并发症时，需电话随访（本研究无这种情况） |
| **结果数据** | **测量结果** | 见后面图表 |

## 方法Methods: Trial Quality：随机方案的产生，分配方案的隐 藏，是否使用盲法，是否存在结果数据的不完整，是 否选择性报告结果，及是否存在其它偏倚

| **随机对照数字的产生**  （对于分配序列的生成尽可能详细地说明分配序列的生成方法） | **RCT**  **-低风险**：计算机随机数字生成器 ，扔硬币，洗牌的卡片和信封，掷骰子，抽签等  **-高风险**：通过奇偶或出生日期产生序列 ，通过入院日期产生序列等  **- Unclear**  **A computer-generated randomization list (1 =experimental group, 2 = control group) was prepared by a third party not associated with the study.** |
| --- | --- |
| Allocation concealment  **分配方案的隐藏**  （分配隐藏尽可能详细地描述隐藏随机分配序列的方法，并陈述隐藏 分配序列的可信度） | **·Yes** 低风险 第三方中心分配，顺序编号、不透明、密封的信封等  ·**Unclear**  **·No** 高风险 使用摊开的随机分配表（如随机序列清单），交替或循环，出生日期等  **The randomization list was contained in a sealed, opaque envelope.** |
| Blinding  **是否使用盲法**  因参与者和实施者了解干预情况而导致实施偏倚 | - Patients Yes No Not stated - Manipulator Yes No Not stated - Outcomes collectors Yes No Not stated - Data analysts Yes No Not stated   **The investigator, anesthesiologists, outcome assessor, patients, and statistician were all blinded to group allocation.** |
| Outcome assessment and measurement  **结果数据的完整性** | **·Yes低风险**：无缺失数据；缺失数据的数目在各干预组相当且各组缺失原因类似；  对二分类变量，与观察事件的发生风险相比，缺失比例不足以影响预估的干预效应；  对连续性结局数据，缺失数据的合理效应规模（均数差或标准均数差）不会大到影响观察的效应规模;  **·No** **高风险**：缺失数据的产生很大可能与真实结局相关, 缺失数据的数目及缺失原因在各干预组相差较大  对二分类变量，与观察事件的发生风险相比，缺失比例足以影响预估的干预效应  对连续性结局数据，缺失数据的合理效应规模（均数差或标准均数差）足以影响观察的效应规模;  ·**Unclear** |
| **是否选择性报告结果** | ·**Yes高风险**不是所有的预先说明的首要结局均被报道 ；一个或多个首要结局为采用预先说明的测量方法、分析方法或数据子集来报道；系统评价感兴趣的一个或多个首要结局报道不全，以至于不能纳入meta分析；  ·**No 低风险**发表的报告包括所有的结局，包括预先说明的结局；  ·**Unsure** |
| **是否存在其它偏倚** | 具有与特殊试验设计相关的潜在偏倚来源，或被指欺诈， 或其它问题 否 |
| Notes  **备注** | 作者分析本文局限性   1、 纳入标准中未对BMI限制，这可能是2位患者阻滞失败的原因。 2、 本文只采用了单一的平面内注射方法 3、 作者在超声引导下只确定了3束臂丛支，没有进行神经刺激。 4、 运动功能的恢复时间通常比感觉功能的恢复时间短。但本研究中感觉功能和运动功能的恢复时间相似，推测这可能与感觉未恢复时肢体麻木和感觉减退影响了患者对运动的主观控制有关（但作者进行了事后分析：在术后随访中，作者发现用绷带、石膏、支架和其他工具固定会影响感觉和运动功能恢复的评估。为了检验这一因素的影响，我们对仅接受手指手术的患者(n = 9)进行了事后分析，发现所有参与者的运动功能恢复都早于感觉功能恢复(0.375%组P = 0.004，0.5%组P ＝0.001)） 5、 我们的研究涉及 6、 样本量有限且为单中心研究 |

## 注意： 对方法学资料除“Yes”、“No”、“Unclear”选项外，还应 提示评价者尽可能引用原文所提供的语句，要尽可能对一些重要细 节进行详细描述。

## Results结果

Comparison:	一是进行的是什 么样的比较，二是每一比较中使用的结局指标有哪些，三是结果资料的类型。

Outcome（结果数据）:

Subcategory（亚组）:

主要结局指标

1、BPB成功率：阻滞成功——即阻滞后30mins感觉+运动阻滞评分≥16分p＞0.05

|  | 0.375% Ropivacaine | 0.5% Ropivacaine | p |
| --- | --- | --- | --- |
| N（人数） | 35 | 34 |  |
| BPB成功率 | 34/35（97%） | 33/34（97%） | p＞0.05 |

次要结局指标（**34** in the 0.375% Ropivacaine group and **33** in the 0.5% Ropivacaine group）

1、阻滞起效时间：p＞0.05	阻滞后感觉+运动阻滞评分=16分时

| （Mean[四分位间距，IQR]） | 0.375% Ropivacaine | 0.5% Ropivacaine | p |
| --- | --- | --- | --- |
| N（人数） | 34 | 33 |  |
| 全阻滞起效时间 | 15[15-20]mins | 15[13-20]mins | P=0.48 |
| 感觉阻滞起效时间 | 15[15-20]mins | 15[13-20]mins | P=0.47 |
| 运动阻滞起效时间 | 10[10-15]mins | 10[10-15]mins | P=0.61 |

感觉阻滞（分级：0-无阻滞；1-镇痛（病人有触觉但无冷觉）；2-麻醉（病人无触觉））

运动阻滞（分级：0-无阻滞；1-轻微or部分麻痹；2-完全麻痹））

2、感觉+运动阻滞持续时间：p＜0.05

| 阻滞持续时间 | 0.375% Ropivacaine | 0.5% Ropivacaine | p |
| --- | --- | --- | --- |
| 感觉（Mean[IQR]） | 455[398-490] | 610[570-655] | P＜0.001 |
| 运动（Mean[IQR]） | 470[409-500] | 625[578-665] | P＜0.001 |

3、感觉+运动阻滞恢复时间：p＜0.05 0.5% Ropivacaine恢复时间更长

4、臂丛主要分支的感觉和运动阻滞（阻滞后30mins内）：p＞0.05

（感觉）

（运动）

5、并发症发生：

|  | 0.375% Ropivacaine | 0.5% Ropivacaine | p |
| --- | --- | --- | --- |
| N（人数） | 34 | 33 |  |
| 并发症 | 0/34 | 3/33 | P＞0.05 |

Note：

提取混杂因素：

基金来源? 未说明

作者得出的关键性结论? **就超声引导下进行CCS BPB的成功率而言，0.375%罗哌卡因不劣于0.5%罗哌卡因。**

作者对混杂因素的评价等? 未评价
