# Supplementary material for: Optimal concentration of ropivacaine for brachial plexus blocks in adult patients undergoing upper limb surgeries: a systematic review and meta-analysis
Source: Front Pharmacol. 2023 Nov 16;14:1288697. doi: 10.3389/fphar.2023.1288697 (PMC10687368; doi:10.3389/fphar.2023.1288697)
Supplement: Supplementary file 3 [file DataSheet1.ZIP › 肌间沟入路 9/0.5% ropivacaine/Andrea Casati1999📕Pulmonary function changes after interscalene brachial plexus anesthesia with 0.5% and 0.75% ropivacaine A double-blinded comparison with 2% mepivacaine.doc]

**附件二：**

**基本信息**

**Date（提取日期）	Reviewer（提取者姓名）: 2023.01.10 吴琳**

**Study Title（文献标题）Pulmonary Function Changes After Interscalene Brachial Plexus Anesthesia with 0.5% and 0.75% Ropivacaine: A Double-Blinded Comparison with 2% Mepivacaine**

| First author**第一作者** | Andrea Casati |
| --- | --- |
| Year of publication **出版年** | 1999 |
| Country of publication **出版国家** | Italy |
| Publication type**出版的类型** | Journal |
| **作者联系方式**  （一些文献能够获取的信息不全，需要联系作者） | casati.andrea@hsr.it |

**纳入文**献的合格性

| **研究是否为随机对照** | 是： ✔ 不是： |
| --- | --- |
| **参与者是否为诊断明确的患者** | ✔ |
| **干预措施是否符合要求** | 是： _✔__；不是：_______ |
| **备注：____________________________________________________________________________**  （如果研究不是随机对照干预文献即排除，注明排除的理由就好） | |

**研究对象的特征（参与者的总人数及各组人 数、人口学信息（性别、年龄等）、研究地点、纳入 人群的基础状况、有无合并症，以及纳入排除标准 等**）

| 研究对象的特征 | | Study |
| --- | --- | --- |
| **参与者的总人数及干预和对照组的各组人 数** | | 30人，10人/组。拟在ISB下进行肩部手术的患者  （1）20ml **0.5%** Ropivacaine  （2）20ml **0.75%** Ropivacaine  （3）20ml 2% Mepivacaine |
| **研究对象** | | - 各组对象基线一致（p＞0.05）   纳入对象基线表（如下表）   |
| **研究对象纳入标准** | | 1、 年龄 18-65岁 2、 ASA I-II级 3、 拟在ISB下行肩关节手术 |
| **研究对象排除标准** | | 1、 呼吸或心脏疾病 2、 糖尿病、周围神经系统疾病 3、 镇痛药物慢性治疗史 |
| **参与者的国家/研究地点** | | 如来自美国的研究，在 社区照顾中心或家庭中生活的老年人 |
| **干预组的措施**  (包括持续的时间) | | （1）**20ml**/组/人。 Winnie法。阻滞失败（需要全麻来完成手术）的患者数据从研究中剔除——无。 |
| **对照组的措施**  (包括持续的时间) | |  |
| **随访时间** | | 出院后一周随访（有无并发症？） |
| **结果数据** | **测量结果** | 见后面图表 |

## 方法Methods: Trial Quality：随机方案的产生，分配方案的隐 藏，是否使用盲法，是否存在结果数据的不完整，是 否选择性报告结果，及是否存在其它偏倚

| **随机对照数字的产生**  （对于分配序列的生成尽可能详细地说明分配序列的生成方法） | **RCT**  **-低风险**：计算机随机数字生成器 ，扔硬币，洗牌的卡片和信封，掷骰子，抽签等  **-高风险**：通过奇偶或出生日期产生序列 ，通过入院日期产生序列等  **- Unclear**  **Using sealed envelopes，patients were randomized to receive 20 mL of either2% mepivacaine (n＝10), 0.5% ropivacaine (n＝10), or0.75% ropivacaine (n＝10)** |
| --- | --- |
| Allocation concealment  **分配方案的隐藏**  （分配隐藏尽可能详细地描述隐藏随机分配序列的方法，并陈述隐藏 分配序列的可信度） | **·Yes** 低风险 第三方中心分配，顺序编号、不透明、密封的信封等  ·**Unclear**  **·No** 高风险 使用摊开的随机分配表（如随机序列清单），交替或循环，出生日期等  **Using sealed envelopes，patients were randomized to receive 20 mL of either2% mepivacaine (n＝10), 0.5% ropivacaine (n＝10), or0.75% ropivacaine (n＝10)** |
| Blinding  **是否使用盲法**  因参与者和实施者了解干预情况而导致实施偏倚 | - Patients Yes No Not stated - Manipulator Yes No Not stated - Outcomes collectors Yes No Not stated - Data analysts Yes No Not stated   **双盲**  **Sterile syringes containing the local anesthetic solution were prepared in a double-blinded fashion by one of the authors, who did not take part in the further management of the patients.** |
| Outcome assessment and measurement  **结果数据的完整性** | **·Yes低风险**：无缺失数据；缺失数据的数目在各干预组相当且各组缺失原因类似；  对二分类变量，与观察事件的发生风险相比，缺失比例不足以影响预估的干预效应；  对连续性结局数据，缺失数据的合理效应规模（均数差或标准均数差）不会大到影响观察的效应规模;  **·No** **高风险**：缺失数据的产生很大可能与真实结局相关, 缺失数据的数目及缺失原因在各干预组相差较大  对二分类变量，与观察事件的发生风险相比，缺失比例足以影响预估的干预效应  对连续性结局数据，缺失数据的合理效应规模（均数差或标准均数差）足以影响观察的效应规模;  ·**Unclear** |
| **是否选择性报告结果** | ·**Yes高风险**不是所有的预先说明的首要结局均被报道 ；一个或多个首要结局为采用预先说明的测量方法、分析方法或数据子集来报道；系统评价感兴趣的一个或多个首要结局报道不全，以至于不能纳入meta分析；  ·**No 低风险**发表的报告包括所有的结局，包括预先说明的结局；  ·**Unsure** |
| **是否存在其它偏倚** | 具有与特殊试验设计相关的潜在偏倚来源，或被指欺诈， 或其它问题 无 |
| Notes  **备注** | 无 |

## 注意： 对方法学资料除“Yes”、“No”、“Unclear”选项外，还应 提示评价者尽可能引用原文所提供的语句，要尽可能对一些重要细 节进行详细描述。

## Results结果

Comparison:	一是进行的是什 么样的比较，二是每一比较中使用的结局指标有哪些，三是结果资料的类型。

Outcome（结果数据）:

Subcategory（亚组）:

主要结局指标、次要结局指标

1、阻滞起效时间（C4-C7皮区无针刺感觉+肩关节不能活动）：

**与0.5% ropivacaine相比， 0.75% ropivacaine起效更快（p＜0.05）**

2、肺功能变化：全部30例患者均有同侧半膈瘫和肺活量平均大幅下降

**总之，肺功能减少方面：两组 P＜0.05（然而，这种不同可能与运动阻滞发生快慢有关，因为局麻药注射后30min，三组肺功能测试无差异。）**

|  | 0.5% ropivacaine | 0.75% ropivacaine | p |
| --- | --- | --- | --- |
| 肺活量下降程度 | 40%±17% | 41%±22% | P＞0.05 |
| 第1s用力呼气量 | 30%±19% | 38%±26% |  |
| FVC-5mins/10mins |  |  | P＜0.05 |
| PEF-5mins/10mins |  |  | P＜0.05 |

3、术后首次需要镇痛药时的疼痛评分（100-mm VAS评分）p＞0.05

术后24h内需要镇痛药情况：

|  | 0.5% ropivacaine | 0.75% ropivacaine | 2%mepivacaine |
| --- | --- | --- | --- |
| 术后24h内需要镇痛药 | 2/20（20%） | 0/10 | 0/10 |

4、阻滞质量和麻醉技术患者接受度 p＞0.05

5、并发症：出院时、出院后1周	无并发症发生

Note：

提取混杂因素：

基金来源? 未说明

作者得出的关键性结论? ISB后30mins，与0.75%罗哌卡因或2%美哌卡因相比，0.5%罗哌卡因在肺功能改变方面没有临床相关优势。但是0.75%罗哌卡因起效时间短，类似于美哌卡因，术后镇痛时间长。因为阻滞起效快和镇痛时间长是局麻的重要目标，故0.75%罗哌卡因用于ISB也许是一个合适的选择。

作者对混杂因素的评价等? 无
