# Supplementary material for: Optimal concentration of ropivacaine for brachial plexus blocks in adult patients undergoing upper limb surgeries: a systematic review and meta-analysis
Source: Front Pharmacol. 2023 Nov 16;14:1288697. doi: 10.3389/fphar.2023.1288697 (PMC10687368; doi:10.3389/fphar.2023.1288697)
Supplement: Supplementary file 3 [file DataSheet1.ZIP › 肌间沟入路 9/0.5% ropivacaine/Ben Safa2021📕Comparison of the Analgesic Duration of 0.5% Bupivacaine With 1200,000 Epinephrine Versus 0.5% Ropivacaine Versus 1% Ropivacaine for Low-Volume Ultrasound-Guided IBPB.doc]

**附件二：**

**基本信息**

**Date（提取日期）	Reviewer（提取者姓名）: 2023.01.06 吴琳**

**Study Title（文献标题）Comparison of the Analgesic Duration of 0.5% Bupivacaine With 1:200,000 Epinephrine Versus 0.5% Ropivacaine Versus 1% Ropivacaine for Low-Volume Ultrasound-Guided Interscalene Brachial Plexus Block: A Randomized Controlled Trial**

| First author**第一作者** | Ben Safa |
| --- | --- |
| Year of publication **出版年** | 2021年 |
| Country of publication **出版国家** | Canada |
| Publication type**出版的类型** | Journal |
| **作者联系方式**  （一些文献能够获取的信息不全，需要联系作者） | stephen.choi@sunnybrook.ca |

**纳入文**献的合格性

| **研究是否为随机对照** | 是： ✔ 不是： |
| --- | --- |
| **参与者是否为诊断明确的患者** | ✔ |
| **干预措施是否符合要求** | 是：_✔_；不是：_______ |
| **备注：____________________________________________________________________________**  （如果研究不是随机对照干预文献即排除，注明排除的理由就好） | |

**研究对象的特征（参与者的总人数及各组人 数、人口学信息（性别、年龄等）、研究地点、纳入 人群的基础状况、有无合并症，以及纳入排除标准 等**）

| 研究对象的特征 | | Study |
| --- | --- | --- |
| **参与者的总人数及干预和对照组的各组人 数** | | 60人（3组）  （1）0.5%Bupivacaine+1:20万NE，5ml  （2）0.5%Ropivacaine，5ml  （3）1%Ropivacaine，5ml   |
| **研究对象** | | - 2016.05-2018.10期间入组的拟行肩关节镜手术的患者 - 各组对象基线一致（p＞0.05）   纳入对象基线表（如下表）   |
| **研究对象纳入标准** | | 1、 年龄 18-80岁 2、 ASA I-III级 3、 拟行肩关节镜手术 |
| **研究对象排除标准** | | 1、 对局麻药过敏/不耐受 2、 (BMI)≥35 kg*m−2 3、 ISB禁忌症： 严重COPD、凝血功能障碍、同侧上肢神经功能缺陷、局部感染 4、 孕妇或哺乳期女性 5、 慢性阿片类药物使用(30mg口服吗啡当量/天) |
| **参与者的国家/研究地点** | | 来自加拿大的研究，拟行肩关节镜手术的患者 |
| **干预组的措施**  (包括持续的时间) | | （1）术前1h：口服氢吗啡酮3mg，对乙酰氨基酚1000 mg (如果体重＜60 kg则650 mg)，口服塞来昔布400 mg (如果体重＜60 kg则200 mg)。术前常规监测，咪达唑仑1–2 mg  （2）ISB：5ml/组。实施ISB后20mins评估运动阻滞。  （3）全麻 |
| **对照组的措施**  (包括持续的时间) | |  |
| **随访时间** | | 电话随访 |
| **结果数据** | **测量结果** | 见后面图表 |

## 方法Methods: Trial Quality：随机方案的产生，分配方案的隐 藏，是否使用盲法，是否存在结果数据的不完整，是 否选择性报告结果，及是否存在其它偏倚

| **随机对照数字的产生**  （对于分配序列的生成尽可能详细地说明分配序列的生成方法） | **RCT：**  **-低风险**：计算机随机数字生成器 ，扔硬币，洗牌的卡片和信封，掷骰子，抽签等  **-高风险**：通过奇偶或出生日期产生序列 ，通过入院日期产生序列等  **- Unclear**  **The computer-generated randomization sequence and sequentially numbered, sealed, opaque envelopes were prepared by an individual not other wise involved in study conduct to maintain blinding and allocation concealment. The randomization list was kept in a secure location inaccessible to investigators, statistician, and outcome assessors.** |
| --- | --- |
| Allocation concealment  **分配方案的隐藏**  （分配隐藏尽可能详细地描述隐藏随机分配序列的方法，并陈述隐藏 分配序列的可信度） | **·Yes** 低风险 第三方中心分配，顺序编号、不透明、密封的信封等  ·**Unclear**  **·No** 高风险 使用摊开的随机分配表（如随机序列清单），交替或循环，出生日期等 |
| Blinding  **是否使用盲法**  因参与者和实施者了解干预情况而导致实施偏倚 | - Patients Yes No Not stated - Manipulator Yes No Not stated - Outcomes collectors Yes No Not stated - Data analysts Yes No Not stated   **全盲All individuals were blinded including study participants, anes**  **thesiologists, surgeons, research personnel, and statistician.** |
| Outcome assessment and measurement  **结果数据的完整性** | **·Yes低风险**：无缺失数据；缺失数据的数目在各干预组相当且各组缺失原因类似；  对二分类变量，与观察事件的发生风险相比，缺失比例不足以影响预估的干预效应；  对连续性结局数据，缺失数据的合理效应规模（均数差或标准均数差）不会大到影响观察的效应规模;  **·No** **高风险**：缺失数据的产生很大可能与真实结局相关, 缺失数据的数目及缺失原因在各干预组相差较大  对二分类变量，与观察事件的发生风险相比，缺失比例足以影响预估的干预效应  对连续性结局数据，缺失数据的合理效应规模（均数差或标准均数差）足以影响观察的效应规模;  ·**Unclear** |
| **是否选择性报告结果** | ·**Yes高风险**不是所有的预先说明的首要结局均被报道 ；一个或多个首要结局为采用预先说明的测量方法、分析方法或数据子集来报道；系统评价感兴趣的一个或多个首要结局报道不全，以至于不能纳入meta分析；  ·**No 低风险**发表的报告包括所有的结局，包括预先说明的结局；  ·**Unsure** |
| **是否存在其它偏倚** | 报告偏倚：the majority of data collection was based on participants' diary/self reporting. |
| Notes  **备注** | 作者报告本文局限性  （1）存在报告偏倚  （2）通过肩外展（运动）的减少（与阻滞前相比）来评估ISB阻滞效果，没有感觉阻滞方面的测量。（其中，肩部本身病变也可能影响肩外展运动，可能影响对阻滞的评估）  （3）但结果减少了选择/检测偏倚，因为研究进行了意向治疗分析(所有进行手术的随机患者)。 |

## 注意： 对方法学资料除“Yes”、“No”、“Unclear”选项外，还应 提示评价者尽可能引用原文所提供的语句，要尽可能对一些重要细 节进行详细描述。

## Results结果

Comparison:	一是进行的是什 么样的比较，二是每一比较中使用的结局指标有哪些，三是结果资料的类型。

Outcome（结果数据）:

Subcategory（亚组）:

主要结局指标

1、阻滞（镇痛）持续时间：即ISB实施结束到患者诉NRS评分＞3分

|  | 0.5% Bupivacaine  +1:20万NE | 0.5% Ropivacaine | 1% Ropivacaine | p |
| --- | --- | --- | --- | --- |
| 阻滞（镇痛）持续时间（小时，h） | 14.1 ± 7.4  （1.7–23） | 13.8 ± 4.5  （7.1-22.5） | 15.8 ± 6.3（1.1–31.5） | P＞0.05 |

次要结局指标（**P＞0.05**）

1、运动阻滞时间：即ISB实施结束到运动功能恢复至基线水平

|  | 0.5% Bupivacaine  +1:20万NE | 0.5% Ropivacaine | 1% Ropivacaine | p |
| --- | --- | --- | --- | --- |
| 运动阻滞时间  （小时，h） | 17.6 h (7.7) | 14.9 h (5.7) | 18.5 h (9.7) | P＞0.05 |

2、首次使用阿片类药物时间：

|  | 0.5% Bupivacaine  +1:20万NE | 0.5% Ropivacaine | 1% Ropivacaine | p |
| --- | --- | --- | --- | --- |
| 首次使用阿片类药物时间（median (IQR)） | 5.6 h (7.9) | 9.4 h (9.5) | 10.4 h (13.2) | P＞0.05 |

3、阿片药物累计消耗量： p＝0.04（但临床意义不大）出院前住院期间、第0天20点、第1天12点、第2天12点

4、NRS评分：第0天20点、第1天12点、第2天12点

5、术后1h房间空气氧饱和度

6、术后恶心呕吐

Note：

提取混杂因素：暂无

基金来源? 未说明

作者得出的关键性结论? **阴性结果。在单次注射低容量US-ISB的情况下，已经证明了相等浓度的罗哌卡因和布比卡因之间疗效相似。此外，将罗哌卡因浓度从0.5%增加到1%并没有延长US-ISB的持续时间**。

作者对混杂因素的评价等? 无
