# Supplementary material for: Optimal concentration of ropivacaine for brachial plexus blocks in adult patients undergoing upper limb surgeries: a systematic review and meta-analysis
Source: Front Pharmacol. 2023 Nov 16;14:1288697. doi: 10.3389/fphar.2023.1288697 (PMC10687368; doi:10.3389/fphar.2023.1288697)
Supplement: Supplementary file 3 [file DataSheet1.ZIP › 肌间沟入路 9/0.5% ropivacaine/Stephen M. Klein1998📕 A comparison of 0.5% bupivacaine, 0.5% ropivacaine, and 0.75% ropivacaine for interscalene brachial plexus block.doc]

**附件二：**

**基本信息**

**Date（提取日期）	Reviewer（提取者姓名）: 2023.01.03 吴琳**

**Study Title（文献标题）A comparison of 0.5% bupivacaine, 0.5% ropivacaine, and 0.75% ropivacaine for interscalene brachial plexus block**

| First author**第一作者** | Stephen M. Klein |
| --- | --- |
| Year of publication **出版年** | 1998 |
| Country of publication **出版国家** | （美国）北卡罗来纳州（North Carolina） |
| Publication type**出版的类型** | Journal |
| **作者联系方式**  （一些文献能够获取的信息不全，需要联系作者） | klein006@mc.duke.edu |

**纳入文**献的合格性

| **研究是否为随机对照** | 是：✔ 不是： |
| --- | --- |
| **参与者是否为诊断明确的患者** | ✔ |
| **干预措施是否符合要求** | 是：_✔_；不是：_______ |
| **备注：____________________________________________________________________________**  （如果研究不是随机对照干预文献即排除，注明排除的理由就好） | |

**研究对象的特征（参与者的总人数及各组人 数、人口学信息（性别、年龄等）、研究地点、纳入 人群的基础状况、有无合并症，以及纳入排除标准 等**）

| 研究对象的特征 | | Study |
| --- | --- | --- |
| **参与者的总人数及干预和对照组的各组人 数** | | 75名成年患者（门诊肩部手术），分成3组：  组1（0.5% bupivacaine，含1:40万肾上腺素）, 25人（-（3+1）人）  组2（ 0.5% ropivacaine，含1:40万肾上腺素）,25人（-3人）  组3（0.75% ropivacaine，含1:40万肾上腺）,25人（-1人） |
| **研究对象** | | - 各组对象基线一致（p＞0.05）   纳入对象基线表（如下表）   |
| **研究对象纳入标准** | | 1、 年龄 ≥18岁 2、 ASA I-III级 3、 知情同意 |
| **研究对象排除标准** | | 1、 凝血障碍 2、 穿刺部位感染   3、手术对侧半膈肌麻痹 |
| **参与者的国家/研究地点** | | 来自美国的研究，在医院门诊进行肩部手术的成年人 |
| **干预组的措施**  (包括持续的时间) | | （1）术前：患者iv咪达唑仑(l-5 mg)和芬太尼(50-250μg)镇静至中度镇静(即可根据命令唤醒)。  （2）30 mL/组；肌间沟入路-臂丛神经阻滞，Winnie法。神经刺激器使肌肉收缩电流＜0.5mA时，以3ml为增量注射30ml罗哌卡因。局麻药注射后每隔1分钟对患者进行评估（评估内容：肩外展（运动）+C5、6皮区针刺（感觉））  （3）记录切皮前：针刺颈丛（C2-4）感觉是否消失  （4）阻滞失败：阻滞30min后仍可肩外展（这些患者在数据分析时剔除）——有7人阻滞失败  （5）感觉、运动阻滞成功后，可进行手术 |
| **对照组的措施**  (包括持续的时间) | |  |
| **随访时间** | | 无随访 |
| **结果数据** | **测量结果** | 见后面图表 |

## 方法Methods: Trial Quality：随机方案的产生，分配方案的隐 藏，是否使用盲法，是否存在结果数据的不完整，是 否选择性报告结果，及是否存在其它偏倚

| **随机对照数字的产生**  （对于分配序列的生成尽可能详细地说明分配序列的生成方法） | **RCT：**  **-低风险**：计算机随机数字生成器 ，扔硬币，洗牌的卡片和信封，掷骰子，抽签等  **-高风险**：通过奇偶或出生日期产生序列 ，通过入院日期产生序列等  **- Unclear** |
| --- | --- |
| Allocation concealment  **分配方案的隐藏**  （分配隐藏尽可能详细地描述隐藏随机分配序列的方法，并陈述隐藏 分配序列的可信度） | **·Yes** 低风险 第三方中心分配，顺序编号、不透明、密封的信封等  ·**Unclear**  **·No** 高风险 使用摊开的随机分配表（如随机序列清单），交替或循环，出生日期等 |
| Blinding  **是否使用盲法**  因参与者和实施者了解干预情况而导致实施偏倚 | - Patients Yes No Not stated - Manipulator Yes No Not stated - Outcomes collectors Yes No Not stated - Data analysts Yes No Not stated   双盲 |
| Outcome assessment and measurement  **结果数据的完整性** | **·Yes低风险**：无缺失数据；缺失数据的数目在各干预组相当且各组缺失原因类似；  对二分类变量，与观察事件的发生风险相比，缺失比例不足以影响预估的干预效应；  对连续性结局数据，缺失数据的合理效应规模（均数差或标准均数差）不会大到影响观察的效应规模;  **·No** **高风险**：缺失数据的产生很大可能与真实结局相关, 缺失数据的数目及缺失原因在各干预组相差较大  对二分类变量，与观察事件的发生风险相比，缺失比例足以影响预估的干预效应  对连续性结局数据，缺失数据的合理效应规模（均数差或标准均数差）足以影响观察的效应规模;  ·**Unclear** |
| **是否选择性报告结果** | ·**Yes高风险**不是所有的预先说明的首要结局均被报道 ；一个或多个首要结局为采用预先说明的测量方法、分析方法或数据子集来报道；系统评价感兴趣的一个或多个首要结局报道不全，以至于不能纳入meta分析；  ·**No 低风险**发表的报告包括所有的结局，包括预先说明的结局；  ·**Unsure** |
| **是否存在其它偏倚** | 具有与特殊试验设计相关的潜在偏倚来源，或被指欺诈， 或其它问题 否_ |
| Notes  **备注** | 无 |

## 注意： 对方法学资料除“Yes”、“No”、“Unclear”选项外，还应 提示评价者尽可能引用原文所提供的语句，要尽可能对一些重要细 节进行详细描述。

## Results结果

Comparison:	一是进行的是什 么样的比较，二是每一比较中使用的结局指标有哪些，三是结果资料的类型。

Outcome（结果数据）:

Subcategory（亚组）:

主要结局指标、次要结局指标

1、3组感觉阻滞持续时间：p＞0.05（无具体数据）

2、3组阻滞（运动+感觉）起效时间和恢复时间：p＞0.05

（1）起效时间（A 运动 B+C 感觉）

| 起效时间（单位：min） | 0.5% Bupivacaine | | 0.5% Ropivacaine | | 0.75% Ropivacaine | |
| --- | --- | --- | --- | --- | --- | --- |
|  | N | Mean±SD | N | Mean±SD | N | Mean±SD |
| 运动阻滞 | 21 | 4.14±3.414 | 22 | 4.64±2.69 | 24 | 3.64±1.94 |
| C5区域感觉阻滞 | 21 | 4.95±3.09 | 22 | 5.77±2.63 | 24 | 5.63±2.74 |
| C6区域感觉阻滞 | 21 | 5.14±3.52 | 22 | 5.73±2.20 | 24 | 5.79±2.55 |

（2）恢复时间（A 感觉 B疼痛发作时间 C切口不适时首次使用镇痛药时间）

【无具体数据呈现】

3、

Note：

提取混杂因素：

基金来源? 未说明

作者得出的关键性结论? 3组无差异

作者对混杂因素的评价等? 未说明
