# Supplementary material for: Optimal concentration of ropivacaine for brachial plexus blocks in adult patients undergoing upper limb surgeries: a systematic review and meta-analysis
Source: Front Pharmacol. 2023 Nov 16;14:1288697. doi: 10.3389/fphar.2023.1288697 (PMC10687368; doi:10.3389/fphar.2023.1288697)
Supplement: Supplementary file 3 [file DataSheet1.ZIP › 肌间沟入路 9/0.5% ropivacaine/Susanne C. Krone2001📕Analgesic effects of low-dose ropivacaine for interscalene brachial plexus block for outpatient shoulder surgery - A dose-finding study.doc]

**附件二：**

**基本信息**

**Date（提取日期）	Reviewer（提取者姓名）: 2023.01.04 吴琳**

**Study Title（文献标题）Analgesic Effects of Low-Dose Ropivacaine for Interscalene Brachial Plexus Block for Outpatient Shoulder Surgery—A Dose-Finding Study**

| First author**第一作者** | Susanne C. Krone |
| --- | --- |
| Year of publication **出版年** | 2001 |
| Country of publication **出版国家** | Canada |
| Publication type**出版的类型** | Journal |
| **作者联系方式**  （一些文献能够获取的信息不全，需要联系作者） | vincentchan@compuserve.com |

**纳入文**献的合格性

| **研究是否为随机对照** | 是： ✔ 不是： |
| --- | --- |
| **参与者是否为诊断明确的患者** | ✔ |
| **干预措施是否符合要求** | 是：__✔_；不是：_______ |
| **备注：____________________________________________________________________________**  （如果研究不是随机对照干预文献即排除，注明排除的理由就好） | |

**研究对象的特征（参与者的总人数及各组人 数、人口学信息（性别、年龄等）、研究地点、纳入 人群的基础状况、有无合并症，以及纳入排除标准 等**）

| 研究对象的特征 | | Study |
| --- | --- | --- |
| **参与者的总人数及干预和对照组的各组人 数** | | **66名（-6名，故60名）**门诊患者（肩关节镜手术）  0.125%Ropivacaine 20人 10ml/人  0. 25%Ropivacaine 20人10ml/人  0.5%Ropivacaine 20人10ml/人 |
| **研究对象** | | - 各组对象基线一致（p＞0.05）   纳入对象基线表（如下表）   |
| **研究对象纳入标准** | | 1、年龄 ≥18岁 |
| **研究对象排除标准** | | 1、 对研究药物过敏 2、 臂丛神经阻滞禁忌症 3、 重大呼吸和神经系统疾病 4、 晚期糖尿病 5、 怀孕 6、 慢性疼痛史 |
| **参与者的国家/研究地点** | | 来自加拿大的研究，在门诊拟行肩关节镜手术的成年患者 |
| **干预组的措施**  (包括持续的时间) | | （1）术前：常规监护，咪达唑仑根据需要以1- 2mg的增量给予抗焦虑  （2）肌间沟入路-Winnie法。10ml/组。神经刺激器使肌肉收缩电流≤0.5mA时，30s内注射10ml罗哌卡因。  （3）术中：常规全麻-propofol (2 ~ 3mg/kg、芬太尼(1.5 μg/kg))、罗库溴铵(0.7 ~ 0.9 mg/kg) |
| **对照组的措施**  (包括持续的时间) | |  |
| **随访时间** | | 出院后48h（电话随访）——内容：阻滞完全消失时间、口服镇痛药消耗量、并发症（恶心呕吐、声嘶、霍纳综合征、呼吸困难） |
| **结果数据** | **测量结果** | 见后面图表 |

## 方法Methods: Trial Quality：随机方案的产生，分配方案的隐 藏，是否使用盲法，是否存在结果数据的不完整，是 否选择性报告结果，及是否存在其它偏倚

| **随机对照数字的产生**  （对于分配序列的生成尽可能详细地说明分配序列的生成方法） | **RCT：randomized by a computer-generated list**  **-低风险**：计算机随机数字生成器 ，扔硬币，洗牌的卡片和信封，掷骰子，抽签等  **-高风险**：通过奇偶或出生日期产生序列 ，通过入院日期产生序列等  **- Unclear** |
| --- | --- |
| Allocation concealment  **分配方案的隐藏**  （分配隐藏尽可能详细地描述隐藏随机分配序列的方法，并陈述隐藏 分配序列的可信度） | **·Yes** 低风险 第三方中心分配，顺序编号、不透明、密封的信封等  ·**Unclear**  **·No** 高风险 使用摊开的随机分配表（如随机序列清单），交替或循环，出生日期等 |
| Blinding  **是否使用盲法**  因参与者和实施者了解干预情况而导致实施偏倚 | - Patients Yes No Not stated - Manipulator Yes No Not stated - Outcomes collectors Yes No Not stated - Data analysts Yes No Not stated   **双盲**  **The study solution was prepared by an independent anesthesiologist**  **not involved in the study or patient care.**  **An investigator un aware of the nature of the study medication performed the following assessments** |
| Outcome assessment and measurement  **结果数据的完整性** | **·Yes低风险**：无缺失数据；缺失数据的数目在各干预组相当且各组缺失原因类似；  对二分类变量，与观察事件的发生风险相比，缺失比例不足以影响预估的干预效应；  对连续性结局数据，缺失数据的合理效应规模（均数差或标准均数差）不会大到影响观察的效应规模;  **·No** **高风险**：缺失数据的产生很大可能与真实结局相关, 缺失数据的数目及缺失原因在各干预组相差较大  对二分类变量，与观察事件的发生风险相比，缺失比例足以影响预估的干预效应  对连续性结局数据，缺失数据的合理效应规模（均数差或标准均数差）足以影响观察的效应规模;  ·**Unclear** |
| **是否选择性报告结果** | ·**Yes高风险**不是所有的预先说明的首要结局均被报道 ；一个或多个首要结局为采用预先说明的测量方法、分析方法或数据子集来报道；系统评价感兴趣的一个或多个首要结局报道不全，以至于不能纳入meta分析；  ·**No 低风险**发表的报告包括所有的结局，包括预先说明的结局；  ·**Unsure** |
| **是否存在其它偏倚** | 具有与特殊试验设计相关的潜在偏倚来源，或被指欺诈， 或其它问题 否 |
| Notes  **备注** | 无 |

## 注意： 对方法学资料除“Yes”、“No”、“Unclear”选项外，还应 提示评价者尽可能引用原文所提供的语句，要尽可能对一些重要细 节进行详细描述。

## Results结果

Comparison:	一是进行的是什 么样的比较，二是每一比较中使用的结局指标有哪些，三是结果资料的类型。

Outcome（结果数据）:

Subcategory（亚组）:

主要结局指标、次要结局指标

1、**术后言语疼痛评分**（VRS，0-10分）-进入PACU后20、30、60、120mins评估

2、**镇痛药消耗情况**：

70%（0.25%和0.5%组）不需要，30%（0.125组）不需要，p＜0.03

|  | Group 0.125%  n=20 | Group 0.25%  n=20 | Group 0.5%  n=20 |
| --- | --- | --- | --- |
| 在PACU需morphine者 | 14/20 | 6/20 | 6/20 |
| 在PACU需morphine者的量 | 7.8±3.3 mg | 6.2±2.2 mg | 6.3±2.2 mg |
| 在PACU首次需morphine时间 | 19 mins | 23 mins | 26 mins |

| 医院不需镇痛者 | Group 0.125%  n=20 | Group 0.25%  n=20 | Group 0.5%  n=20 |
| --- | --- | --- | --- |
| 随访人数 | 16（-4人） | 18（-2人） | 17（-3人） |
| 在家首次服用镇痛药时间 | （674±55）mins | （613±241）mins | （649±248）mins |

3、**运动和感觉阻滞**-进入PACU后20、30、60、120mins评估

（1）感觉：C4到T1的皮区使用23号针对针刺感觉进行评估，并将其分为无感觉、减弱或正常。

|  | Group 0.125%  n=20 | Group 0.25%  n=20 | Group 0.5%  n=20 |
| --- | --- | --- | --- |
| 感觉-减弱 | 7/20（35%） | 18/20（90%） | 18/20（90%） |
| 肘部远端 运动-减弱（p＜0.05） | 0/20（0%） | 3/20（15%） | 5/20（25%） |

（2）肌肉运动：C5、C6、C7、C8/T1，将其分为无运动、减弱或正常。

4、**患者在医院使用吗啡、在家中使用酮咯酸or泰诺#3的总量**（见Table 2）

Note：

提取混杂因素：

基金来源? 未提及

作者得出的关键性结论? **小剂量罗哌卡因10 mL 0.25%和0.5%**进行肌间沟臂丛间阻滞，为关节镜手术后的大多数患者提供有效持久的肩部镇痛。

作者对混杂因素的评价等? 无
