# Supplementary material for: Optimal concentration of ropivacaine for brachial plexus blocks in adult patients undergoing upper limb surgeries: a systematic review and meta-analysis
Source: Front Pharmacol. 2023 Nov 16;14:1288697. doi: 10.3389/fphar.2023.1288697 (PMC10687368; doi:10.3389/fphar.2023.1288697)
Supplement: Supplementary file 3 [file DataSheet1.ZIP › 肌间沟入路 9/0.5% ropivacaine/Wenwen Zha2016📕Effects of a fixed low-dose ropivacaine with different volume and concentrations on interscalene brachial plexus block a randomized controlled trial.doc]

**附件二：**

**基本信息**

**Date（提取日期）	Reviewer（提取者姓名）: 2023.01.09 吴琳**

**Study Title（文献标题）Effects of a fixed low-dose ropivacaine with different volume and concentrations on interscalene brachial plexus block: a randomized controlled trial**

| First author**第一作者** | Wenwen Zha |
| --- | --- |
| Year of publication **出版年** | 2016 |
| Country of publication **出版国家** | China |
| Publication type**出版的类型** | Journal |
| **作者联系方式**  （一些文献能够获取的信息不全，需要联系作者） | [liminanesth@aliyun.com](mailto:liminanesth@aliyun.com);  howang@med.wayne.edu |

**纳入文**献的合格性

| **研究是否为随机对照** | 是：✔ 不是： |
| --- | --- |
| **参与者是否为诊断明确的患者** | ✔ |
| **干预措施是否符合要求** | 是：✔；不是：_______ |
| **备注：____________________________________________________________________________**  （如果研究不是随机对照干预文献即排除，注明排除的理由就好） | |

**研究对象的特征（参与者的总人数及各组人 数、人口学信息（性别、年龄等）、研究地点、纳入 人群的基础状况、有无合并症，以及纳入排除标准 等**）

| 研究对象的特征 | | Study |
| --- | --- | --- |
| **参与者的总人数及干预和对照组的各组人 数** | | **99名**，拟在ISB下行肩关节镜手术的患者  （1）0.75% Ropivacaine 6.7ml  （2）0.5% Ropivacaine 10ml  （3）0.25% Ropivacaine 20ml   |
| **研究对象** | | - 2013.12-2014.02入组，拟在ISB下行肩关节镜手术的患者 - 各组对象基线一致（p＞0.05）   纳入对象基线表（如下表）   |
| **研究对象纳入标准** | | 1、 年龄 18-80岁 2、 ASA I-II级 3、 拟行肩关节镜手术 4、 知情同意 |
| **研究对象排除标准** | | 1、 患者拒绝 2、 神经系统或神经肌肉疾病 3、 严重的支气管肺疾病 4、 凝血功能障碍 5、 注射部位感染 6、 已知对研究方案中使用的一种或多种药物过敏 7、 BMI ＞32 kg/m2 8、 服用阿片类镇痛药的患者 |
| **参与者的国家/研究地点** | | 来自中国的研究，在北京大学第三医院拟行肩关节镜手术的患者 |
| **干预组的措施**  (包括持续的时间) | | （1）感觉+运动阻滞： 每5mins评估一次，评估30mins的  ①感觉阻滞：0-感觉正常；1-感觉阻滞（镇痛）；2-感觉消失（麻醉）  阻滞失败：ISB操作完成后30mins内，分级未达2。  ②运动阻滞：0-运动正常；1-运动减弱；2-无运动 |
| **对照组的措施**  (包括持续的时间) | |  |
| **随访时间** | | 如果出现晚期并发症则电话随访（未出现） |
| **结果数据** | **测量结果** | 见后面图表 |

## 方法Methods: Trial Quality：随机方案的产生，分配方案的隐 藏，是否使用盲法，是否存在结果数据的不完整，是 否选择性报告结果，及是否存在其它偏倚

| **随机对照数字的产生**  （对于分配序列的生成尽可能详细地说明分配序列的生成方法） | **RCT：**  **-低风险**：计算机随机数字生成器 ，扔硬币，洗牌的卡片和信封，掷骰子，抽签等  **-高风险**：通过奇偶或出生日期产生序列 ，通过入院日期产生序列等  **- Unclear**  **Using a computer generated random number table，patients were randomized into three groups** |
| --- | --- |
| Allocation concealment  **分配方案的隐藏**  （分配隐藏尽可能详细地描述隐藏随机分配序列的方法，并陈述隐藏 分配序列的可信度） | **·Yes** 低风险 第三方中心分配，顺序编号、不透明、密封的信封等  ·**Unclear**  **·No** 高风险 使用摊开的随机分配表（如随机序列清单），交替或循环，出生日期等  **Sealed opaque envelopes with the study group allocation were opened before the blocks were performed.** |
| Blinding  **是否使用盲法**  因参与者和实施者了解干预情况而导致实施偏倚 | - Patients Yes No Not stated - Manipulator Yes No Not stated - Outcomes collectors Yes No Not stated - Data analysts Yes No Not stated   **双盲**  **An independent anesthesiologist who was not involved in the study prepared the study solution.**  **After completion of the block, all the evaluations were performed by another blinded independent observer who was not involved in grouping, study solution preparing and interscalene block performing.**  **All patients were blind to their group throughout the study.** |
| Outcome assessment and measurement  **结果数据的完整性** | **·Yes低风险**：无缺失数据；缺失数据的数目在各干预组相当且各组缺失原因类似；  对二分类变量，与观察事件的发生风险相比，缺失比例不足以影响预估的干预效应；  对连续性结局数据，缺失数据的合理效应规模（均数差或标准均数差）不会大到影响观察的效应规模;  **·No** **高风险**：缺失数据的产生很大可能与真实结局相关, 缺失数据的数目及缺失原因在各干预组相差较大  对二分类变量，与观察事件的发生风险相比，缺失比例足以影响预估的干预效应  对连续性结局数据，缺失数据的合理效应规模（均数差或标准均数差）足以影响观察的效应规模;  ·**Unclear** |
| **是否选择性报告结果** | ·**Yes高风险**不是所有的预先说明的首要结局均被报道 ；一个或多个首要结局为采用预先说明的测量方法、分析方法或数据子集来报道；系统评价感兴趣的一个或多个首要结局报道不全，以至于不能纳入meta分析；  ·**No 低风险**发表的报告包括所有的结局，包括预先说明的结局；  ·**Unsure** |
| **是否存在其它偏倚** | 具有与特殊试验设计相关的潜在偏倚来源，或被指欺诈， 或其它问题 无 |
| Notes  **备注** | **作者分析本文局限性**   1、 ISB实施是由一位经验丰富的麻醉师做的。因此，我们的研究应该是谨慎地应用到其他情况，因为这是一个低剂量研究，其中一组为6.7 ml的罗哌 2、 本文是应用罗哌卡因得到的结果。对其他局麻药不一定适用 3、 人群不同，结果不一定适用 4、 虽然男性和女性患者比例的差异无统计学意义，但在病人的群体中，患者性别分布是不平衡的，因此性别产生影响不能排除 5、 虽然超声引导下ISB是一个常用方法，来成像臂丛及引导穿刺针插入，该技术存在对与中斜角肌相关的臂丛神经小分支的损伤风险，如肩胛背N和胸长神经。 |

## 注意： 对方法学资料除“Yes”、“No”、“Unclear”选项外，还应 提示评价者尽可能引用原文所提供的语句，要尽可能对一些重要细 节进行详细描述。

## Results结果

Comparison:	一是进行的是什 么样的比较，二是每一比较中使用的结局指标有哪些，三是结果资料的类型。

Outcome（结果数据）:

Subcategory（亚组）:

一、主要结局指标

1、感觉+运动阻滞起效时间（即ISB操作结束到针刺C5-C6皮区分级达2）

p＜0.05。浓度越高，起效越快

| 阻滞起效时间 | 0.75% Ropivacaine | 0.5% Ropivacaine | 0.25% Ropivacaine | p |
| --- | --- | --- | --- | --- |
| 感觉（min）  Median（range） | N=33 | N=29 | N=33 |  |
|  | 5 (5–20) | 10 (5–20) | 20 (5–30) | p=0.000 |
| 运动（min）Median（range） | N=33 | N=28 | N=13 |  |
|  | 10 (5–25) | 15 (5–30) |  | p=0.000 |

二、次要结局指标

1、术后疼痛评分（NRPS量表，0-10分） p＞0.05

2、疼痛最强评分p＞0.05

3、疼痛最强时超过4分的比例p＞0.05

4、睡眠质量（阻滞后24h评估）：0 =无疼痛引起的睡眠障碍，1 =存在疼痛引起的睡眠障碍p＞0.05

5、阻滞满意度（阻滞后24h评估）p＞0.05

6、运动阻滞起效时间 p＜0.05

7、阻滞成功率 p＞0.05

8、镇痛药需要量 p＞0.05

9、阻滞侧手握力 p＞0.05

| 阻滞侧手握力 | 0.75% Ropivacaine | 0.5% Ropivacaine | 0.25% Ropivacaine | p |
| --- | --- | --- | --- | --- |
| 术前 | N=33 | N=29 | N=33 |  |
|  | 24.0 ± 11.5 kg | 27.6 ± 12.9 kg | 26.6 ± 9.6 kg | P＞0.05 |

| 阻滞侧手握力 | 0.75% Ropivacaine | 0.5% Ropivacaine | 0.25% Ropivacaine |
| --- | --- | --- | --- |
| 人数 | N=33 | N=29 | N=33 |
| 术前（T0） | 100% | 100% | 100% |
| PACU（T1） | 41% | 32% | 36% |
| ISB后4h（T2） | 48% | 46% | 42% |
| ISB后8h（T3） | 70% | 52% | 57% |
| ISB后24h（T4） | 98% | 95% | 91% |

10、半膈肌麻痹的发生率p＞0.05

11、并发症发生率p＞0.05 0.25% Ropivacaine 1人在ISB后30mins发生霍纳综合征

12、基线横膈运动：ISB 30mins后 p＜0.05

| 横膈运动 | 0.75% Ropivacaine | 0.5% Ropivacaine | 0.25% Ropivacaine | p |
| --- | --- | --- | --- | --- |
| 缺失或矛盾 | 19/33（58%） | 20/29（69%） | 23/33（70%） | p=0.000 |

Note：

提取混杂因素：

基金来源? 未说明

作者得出的关键性结论? **Ropivacaine 0.25、0.5、0.75%溶液（50mg）用于ISB后肩关节镜手术可产生类似的阻滞，副作用小。并且0.75% Ropivacaine似乎更可取，因为它阻滞的起效时间更快。**

作者对混杂因素的评价等? 无
