# Supplementary material for: Optimal concentration of ropivacaine for brachial plexus blocks in adult patients undergoing upper limb surgeries: a systematic review and meta-analysis
Source: Front Pharmacol. 2023 Nov 16;14:1288697. doi: 10.3389/fphar.2023.1288697 (PMC10687368; doi:10.3389/fphar.2023.1288697)
Supplement: Supplementary file 3 [file DataSheet1.ZIP › 肌间沟入路 9/other concentration/Andrew K. Wong2016📕 Effect of Local Anesthetic Concentration (0.2% vs 0.1% 罗哌) on Pulmonary Function, and Analgesia After 超声引导下 Interscalene Brachial Plexus Block A Randomized Controlled Study.doc]

**附件二：**

**基本信息**

**Date（提取日期）	Reviewer（提取者姓名）: 2023.01.08**

**Study Title（文献标题）Effect of Local Anesthetic Concentration (0.2% vs 0.1% Ropivacaine) on Pulmonary Function,and Analgesia After Ultrasound-Guided Interscalene Brachial Plexus Block:A Randomized Controlled Study**

| First author**第一作者** | Andrew K. Wong |
| --- | --- |
| Year of publication **出版年** | 2016 |
| Country of publication **出版国家** | USA |
| Publication type**出版的类型** | Journal |
| **作者联系方式**  （一些文献能够获取的信息不全，需要联系作者） | nabil.elkassabany@uphs.upenn.edu. |

**纳入文**献的合格性

| **研究是否为随机对照** | 是：✔ 不是： |
| --- | --- |
| **参与者是否为诊断明确的患者** | ✔ |
| **干预措施是否符合要求** | 是：✔ ___；不是：_______ |
| **备注：____________________________________________________________________________**  （如果研究不是随机对照干预文献即排除，注明排除的理由就好） | |

**研究对象的特征（参与者的总人数及各组人 数、人口学信息（性别、年龄等）、研究地点、纳入 人群的基础状况、有无合并症，以及纳入排除标准 等**）

| 研究对象的特征 | | Study |
| --- | --- | --- |
| **参与者的总人数及干预和对照组的各组人 数** | | **50人**，拟行肩关节镜手术的患者  （1）0.1% Ropivacaine  （2）0.2% Ropivacaine   |
| **研究对象** | | - 2014年9-12月入组，拟在ISB下行肩关节镜手术的患者 - 各组对象基线一致（p＞0.05）   纳入对象基线表（如下表）   |
| **研究对象纳入标准** | | 1、 年龄 18-75岁 2、 ASA I-III级 3、 拟行肩关节镜手术 4、 精神正常 5、 知情同意 |
| **研究对象排除标准** | | 1、 对局麻药或其他研究药物过敏 2、 凝血功能障碍 3、 慢性阿片类药物使用(术前6个月以上) 4、 既往患有慢性阻塞性肺疾病(COPD)或严重哮喘 5、 已知膈肌或肋间肌功能障碍 6、 影响呼吸的神经肌肉疾病 7、 BMI＞40kg/m2 8、 不能说或理解英语 |
| **参与者的国家/研究地点** | | 来自美国的研究，拟在ISB下行肩关节镜手术的患 |
| **干预组的措施**  (包括持续的时间) | | （1）**20ml/组/人**。  ISB成功：手臂及肩部的感觉与运动功能消失。  （2）全麻常规。 |
| **对照组的措施**  (包括持续的时间) | |  |
| **随访时间** | | 电话随访：术后24h |
| **结果数据** | **测量结果** | 见后面图表 |

## 方法Methods: Trial Quality：随机方案的产生，分配方案的隐 藏，是否使用盲法，是否存在结果数据的不完整，是 否选择性报告结果，及是否存在其它偏倚

| **随机对照数字的产生**  （对于分配序列的生成尽可能详细地说明分配序列的生成方法） | **RCT：**  **-低风险**：计算机随机数字生成器 ，扔硬币，洗牌的卡片和信封，掷骰子，抽签等  **-高风险**：通过奇偶或出生日期产生序列 ，通过入院日期产生序列等  **- Unclear**  Study participants were randomized using a computer-generated randomization sequence. |
| --- | --- |
| Allocation concealment  **分配方案的隐藏**  （分配隐藏尽可能详细地描述隐藏随机分配序列的方法，并陈述隐藏 分配序列的可信度） | **·Yes** 低风险 第三方中心分配，顺序编号、不透明、密封的信封等  ·**Unclear**  **·No** 高风险 使用摊开的随机分配表（如随机序列清单），交替或循环，出生日期等  Group allocation was written in a sealed envelope that was opened after the patient consented for the study. |
| Blinding  **是否使用盲法**  因参与者和实施者了解干预情况而导致实施偏倚 | - Patients Yes No Not stated - Manipulator Yes No Not stated - Outcomes collectors Yes No Not stated - Data analysts Yes No Not stated   **双盲**  **Patients and research assistants assessing nerve blockade and diaphragmatic function were blinded to the treatment allocation.** |
| Outcome assessment and measurement  **结果数据的完整性** | **·Yes低风险**：无缺失数据；缺失数据的数目在各干预组相当且各组缺失原因类似；  对二分类变量，与观察事件的发生风险相比，缺失比例不足以影响预估的干预效应；  对连续性结局数据，缺失数据的合理效应规模（均数差或标准均数差）不会大到影响观察的效应规模;  **·No** **高风险**：缺失数据的产生很大可能与真实结局相关, 缺失数据的数目及缺失原因在各干预组相差较大  对二分类变量，与观察事件的发生风险相比，缺失比例足以影响预估的干预效应  对连续性结局数据，缺失数据的合理效应规模（均数差或标准均数差）足以影响观察的效应规模;  ·**Unclear** |
| **是否选择性报告结果** | ·**Yes高风险**不是所有的预先说明的首要结局均被报道 ；一个或多个首要结局为采用预先说明的测量方法、分析方法或数据子集来报道；系统评价感兴趣的一个或多个首要结局报道不全，以至于不能纳入meta分析；  ·**No 低风险**发表的报告包括所有的结局，包括预先说明的结局；  ·**Unsure** |
| **是否存在其它偏倚** | 具有与特殊试验设计相关的潜在偏倚来源，或被指欺诈， 或其它问题 无 |
| Notes  **备注** | **作者说明本文局限性**   1、 首先，排除BMI较高的患者和肺部疾病患者可能会限制研究结果对横膈膜麻痹影响最大的患者队列的普遍性。 2、 第二，尽管研究者尽最大的努力并每天多次尝试与患者联系，但就疼痛日记的结果而言，许多患者未能进行随访。 3、 第三，由于手术室提前可用，我们未对14例患者在阻滞30mins后进行评估。 |

## 注意： 对方法学资料除“Yes”、“No”、“Unclear”选项外，还应 提示评价者尽可能引用原文所提供的语句，要尽可能对一些重要细 节进行详细描述。

## Results结果

Comparison:	一是进行的是什 么样的比较，二是每一比较中使用的结局指标有哪些，三是结果资料的类型。

Outcome（结果数据）:

Subcategory（亚组）:

主要结局指标、次要结局指标

1、膈移动（M型超声评估）：ISB前、ISB后30mins、术后30-45mins

2、肺功能（便携式肺活量计评估）：FVC、FEV1、PEF—— ISB前、ISB后30mins、术后30mins

3、在PACU的氧饱（降低：指尖氧饱＜93%）：没有病人出现氧饱降低

4、	PACU停留时长 p＞0.05

疼痛评分（DVPRS）——PACU后30mins和60mins	p＞0.05

在PACU镇痛药需要量 p＞0.05

感觉、运动阻滞持续时间 p=0.04

ISB后72h阿片类药物使用量 p=0.02

总阿片类药物消耗量 p＞0.05

疼痛评分曲线下面积（POD0-POD3）p＞0.05

5、术后24h恢复质量评分（Quality of recovery score，QOR）p＞0.05

|  | 0.1% Ropivacaine | 0.2% Ropivacaine | p |
| --- | --- | --- | --- |
| 术后24h恢复质量评分 | 15 (13–17) | 14 (13–17) | P =0.7 |

Note：

提取混杂因素：

基金来源? 未说明

作者得出的关键性结论? **总之，本研究发现，在超声引导下使用0.1% Ropivacaine ISB可在肩关节镜术后即刻提供足够的镇痛。0.1% Ropivacaine损害肺功能程度低于0.2% Ropivacaine。0.2% Ropivacaine阻滞持续时间更长且在ISB 72h后对阿片类药物消耗量更少。**

作者对混杂因素的评价等? 未说明
