# Supplementary material for: Optimal concentration of ropivacaine for brachial plexus blocks in adult patients undergoing upper limb surgeries: a systematic review and meta-analysis
Source: Front Pharmacol. 2023 Nov 16;14:1288697. doi: 10.3389/fphar.2023.1288697 (PMC10687368; doi:10.3389/fphar.2023.1288697)
Supplement: Supplementary file 3 [file DataSheet1.ZIP › 肌间沟入路 9/other concentration/Michael J. Fredrickson2012📕Randomized Study of The effect of local anaesthetic volume and concentration on the duration of peripheral nerve blockade in patients having shoulder surgery.doc]

**附件二：**

**基本信息**

**Date（提取日期）	Reviewer（提取者姓名）: 2023.01.10 吴琳**

**Study Title（文献标题）Randomized Study of the Effect of Local Anesthetic Volume and Concentration on the Duration of Peripheral Nerve Blockade**

| First author**第一作者** | Michael J. Fredrickson |
| --- | --- |
| Year of publication **出版年** | 2012年 |
| Country of publication **出版国家** | New Zealand |
| Publication type**出版的类型** | Journal |
| **作者联系方式**  （一些文献能够获取的信息不全，需要联系作者） | michaelfredrickson@yahoo.com |

**纳入文**献的合格性

| **研究是否为随机对照** | 是：✔ 不是： |
| --- | --- |
| **参与者是否为诊断明确的患者** | ✔ |
| **干预措施是否符合要求** | 是：_✔__；不是：_______ |
| **备注：____________________________________________________________________________**  （如果研究不是随机对照干预文献即排除，注明排除的理由就好） | |

**研究对象的特征（参与者的总人数及各组人 数、人口学信息（性别、年龄等）、研究地点、纳入 人群的基础状况、有无合并症，以及纳入排除标准 等**）

| 研究对象的特征 | | Study |
| --- | --- | --- |
| **参与者的总人数及干预和对照组的各组人 数** | | 185人，5组。  （1）5ml 0.75%Ropivacaine：40人。数据分析时排除了  （2）10 ml 0.75%Ropivacaine：41人  （3）20 ml 0.75%Ropivacaine：33人  （4）20ml 0.375%Ropivacaine：35人  （5）40 mL0.375%Ropivacaine：36人 |
| **研究对象** | | - 2011.02-2011.12入组 - 各组对象基线一致（p＞0.05）   纳入对象基线表（如下表）   |
| **研究对象纳入标准** | | 1、 年龄 16-80岁 2、 ASA I-III级 3、 在南十字路口(M.J.F.)和北港(A.A.)医院拟行肩部手术 4、 知情同意 |
| **研究对象排除标准** | | 1、 拒绝臂丛神经阻滞 2、 已知涉及术侧手臂的神经疾病 3、 对酰胺类麻醉药过敏 4、 术前接受阿片类药物治疗1个月以上 |
| **参与者的国家/研究地点** | | 来自新西兰的研究，在医院拟在ISB下行肩部手术的患者 |
| **干预组的措施**  (包括持续的时间) | | （1）5ml 0.75%Ropivacaine：40人。数据分析时排除了  （2）10 ml 0.75%Ropivacaine：41人  （3）20 ml 0.75%Ropivacaine：33人  （4）20ml 0.375%Ropivacaine：35人  （5）40 mL0.375%Ropivacaine：36人 |
| **对照组的措施**  (包括持续的时间) | |  |
| **随访时间** | | 术后24h电话随访：首次疼痛时间时间、镇痛药消耗量、疼痛程度(最严重和“平均”)、手部麻木/运动、最初24h满意度、24h并发症 |
| **结果数据** | **测量结果** | 见后面图 |

## 方法Methods: Trial Quality：随机方案的产生，分配方案的隐 藏，是否使用盲法，是否存在结果数据的不完整，是 否选择性报告结果，及是否存在其它偏倚

| **随机对照数字的产生**  （对于分配序列的生成尽可能详细地说明分配序列的生成方法） | **RCT**  **-低风险**：计算机随机数字生成器 ，扔硬币，洗牌的卡片和信封，掷骰子，抽签等  **-高风险**：通过奇偶或出生日期产生序列 ，通过入院日期产生序列等  **- Unclear**  **Using a computer-generated random number in blocks of 20**  **(www.random.org) random assignment to the 5 groups was implemented by a research assistant remote from the study procedures.** |
| --- | --- |
| Allocation concealment  **分配方案的隐藏**  （分配隐藏尽可能详细地描述隐藏随机分配序列的方法，并陈述隐藏 分配序列的可信度） | **·Yes** 低风险 第三方中心分配，顺序编号、不透明、密封的信封等  ·**Unclear**  **·No** 高风险 使用摊开的随机分配表（如随机序列清单），交替或循环，出生日期等  **Group concealment was by 185 preprepared, sealed opaque**  **envelopes, opened immediately after catheter placement.** |
| Blinding  **是否使用盲法**  因参与者和实施者了解干预情况而导致实施偏倚 | - Patients Yes No Not stated - Manipulator Yes No Not stated - Outcomes collectors Yes No Not stated - Data analysts Yes No Not stated   **The design was a dual-center, prospective, randomized, observer blinded trial.** |
| Outcome assessment and measurement  **结果数据的完整性** | **·Yes低风险**：无缺失数据；缺失数据的数目在各干预组相当且各组缺失原因类似；  对二分类变量，与观察事件的发生风险相比，缺失比例不足以影响预估的干预效应；  对连续性结局数据，缺失数据的合理效应规模（均数差或标准均数差）不会大到影响观察的效应规模;  **·No** **高风险**：缺失数据的产生很大可能与真实结局相关, 缺失数据的数目及缺失原因在各干预组相差较大  对二分类变量，与观察事件的发生风险相比，缺失比例足以影响预估的干预效应  对连续性结局数据，缺失数据的合理效应规模（均数差或标准均数差）足以影响观察的效应规模;  ·**Unclear** |
| **是否选择性报告结果** | ·**Yes高风险**不是所有的预先说明的首要结局均被报道 ；一个或多个首要结局为采用预先说明的测量方法、分析方法或数据子集来报道；系统评价感兴趣的一个或多个首要结局报道不全，以至于不能纳入meta分析；  ·**No 低风险**发表的报告包括所有的结局，包括预先说明的结局；  ·**Unsure** |
| **是否存在其它偏倚** | 具有与特殊试验设计相关的潜在偏倚来源，或被指欺诈， 或其它问题 否 |
| Notes  **备注** | 作者分析  1、本研究次要结局指标中，未有充分效能检出其各组的差异性。 |

## 注意： 对方法学资料除“Yes”、“No”、“Unclear”选项外，还应 提示评价者尽可能引用原文所提供的语句，要尽可能对一些重要细 节进行详细描述。

## Results结果

Comparison:	一是进行的是什 么样的比较，二是每一比较中使用的结局指标有哪些，三是结果资料的类型。

Outcome（结果数据）:

Subcategory（亚组）:

主要结局指标

1、术后首次出现疼痛的时间（5ml组因阻滞失败率高，不进行比较） **p＜0.05**

| Figure 4 | 20 ml 0.75%Ropivacaine | 20 ml  0.5%Ropivacaine | 20 ml 0.375%Ropivacaine | p |
| --- | --- | --- | --- | --- |
| Time to first pain | 13.75（四分位距，10.5-21） | 11.5（四分位距，10-16.75） | 10.75（四分位距，9.75-14） |  |

2、阻滞持续时间

|  | 20 ml 0.75%Ropivacaine | 20 ml 0.375%Ropivacaine | p |
| --- | --- | --- | --- |
| 阻滞持续时间 | 13.75（10.5-21.0） | 10.75（9.75-14.0） | P＜0.05 |

次要结局指标（阻滞效果）-可能检验效能不足

1、镇痛药消耗量 p＞0.05

2、罗哌卡因泵注量 p＞0.05

3、最强肩部NRS评分p＞0.05

4、平均肩部NRS评分p＞0.05

5、手部感觉NRS p＞0.05

6、手部运动NRS p＞0.05

7、第一个24h并发症（呼吸困难）p＞0.05

8、NRS满意度p＞0.05

Note：

提取混杂因素：未说明

基金来源? This work was funded by a grant of US $8000 from the I-Flow Corporation,Lake Forest, CA.

作者得出的关键性结论? **Block duration is influenced by both local anesthetic volume and concentration。**

作者对混杂因素的评价等? 无
