# Supplementary material for: Optimal concentration of ropivacaine for brachial plexus blocks in adult patients undergoing upper limb surgeries: a systematic review and meta-analysis
Source: Front Pharmacol. 2023 Nov 16;14:1288697. doi: 10.3389/fphar.2023.1288697 (PMC10687368; doi:10.3389/fphar.2023.1288697)
Supplement: Supplementary file 3 [file DataSheet1.ZIP › 臂丛入路不明 0/The efficacy of pediatric ultrasound guided brachial plexus block anesthesia and determination of optimal anesthetic drug dosage.doc]

**附件二：**

**基本信息**

**Date（提取日期）	Reviewer（提取者姓名）: 2023.01.03 吴琳**

**Study Title（文献标题）The efficacy of pediatric ultrasound guided brachial plexus block anesthesia and determination of optimal anesthetic drug dosage**

| First author**第一作者** | Chuanguo XU |
| --- | --- |
| Year of publication **出版年** | 2016 |
| Country of publication **出版国家** | China |
| Publication type**出版的类型** | Journal |
| **作者联系方式**  （一些文献能够获取的信息不全，需要联系作者） | wang_guanglei1@163.com |

**纳入文**献的合格性

| **研究是否为随机对照** | 是：✔ 不是： |
| --- | --- |
| **参与者是否为诊断明确的患者** | ✔ |
| **干预措施是否符合要求** | 是：_✔_；不是：_______ |
| **备注：____________________________________________________________________________**  （如果研究不是随机对照干预文献即排除，注明排除的理由就好） | |

**研究对象的特征（参与者的总人数及各组人 数、人口学信息（性别、年龄等）、研究地点、纳入 人群的基础状况、有无合并症，以及纳入排除标准 等**）

| 研究对象的特征 | | Study |
| --- | --- | --- |
| **参与者的总人数及干预和对照组的各组人 数** | | 97人 分成3组：A组32人0.3%罗哌、B组35人0.4%罗哌、C组30人0.5%罗哌 |
| **研究对象基本信息** | | - 2015.1-2015.12，首次收治需要择期上肢手术的患儿 - 各组对象基线一致（p＞0.05）    |
| **研究对象纳入标准** | | 1. 年龄3-14岁  2. ASA I-II级  3.手术时长＞40min  4.没有发育异常 |
| **研究对象排除标准** | | 1. 穿刺部位炎症;  2. 气胸或上肢神经功能障碍  3. 局麻药过敏史  4.合并其他疾病 |
| **参与者的国家/研究地点** | | 如来自中国泰安市妇幼保健院的研究——需要进行上肢手术，进行超声引导下臂丛神经阻滞的患者 |
| **干预组的措施**  (包括持续的时间) | | 24ml罗哌卡因/组。 |
| **对照组的措施**  (包括持续的时间) | | 24ml罗哌卡因/组。 |
| **随访时间** | | 未随访 |
| **结果数据** | **测量结果** | 见后面图表 |

## 方法Methods: Trial Quality：随机方案的产生，分配方案的隐 藏，是否使用盲法，是否存在结果数据的不完整，是 否选择性报告结果，及是否存在其它偏倚

| **随机对照数字的产生**  （对于分配序列的生成尽可能详细地说明分配序列的生成方法） | **RCT：**   - **低风险**：计算机随机数字生成器 ，扔硬币，洗牌的卡片和信封，掷骰子，抽签等 - **高风险**：通过奇偶或出生日期产生序列 ，通过入院日期产生序列等 - **Unclear** |
| --- | --- |
| Allocation concealment  **分配方案的隐藏**  （分配隐藏尽可能详细地描述隐藏随机分配序列的方法，并陈述隐藏 分配序列的可信度） | **·Yes** 低风险 第三方中心分配，顺序编号、不透明、密封的信封等  ·**Unclear**  **·No** 高风险 使用摊开的随机分配表（如随机序列清单），交替或循环，出生日期等 |
| Blinding  **是否使用盲法**  因参与者和实施者了解干预情况而导致实施偏倚 | - Patients Yes No Not stated - Manipulator Yes No Not stated - Outcomes collectors Yes No Not stated - Data analysts Yes No Not stated |
| Outcome assessment and measurement  **结果数据的完整性** | **·Yes低风险**：无缺失数据；缺失数据的数目在各干预组相当且各组缺失原因类似；  对二分类变量，与观察事件的发生风险相比，缺失比例不足以影响预估的干预效应；  对连续性结局数据，缺失数据的合理效应规模（均数差或标准均数差）不会大到影响观察的效应规模;  **·No** **高风险**：缺失数据的产生很大可能与真实结局相关, 缺失数据的数目及缺失原因在各干预组相差较大  对二分类变量，与观察事件的发生风险相比，缺失比例足以影响预估的干预效应  对连续性结局数据，缺失数据的合理效应规模（均数差或标准均数差）足以影响观察的效应规模;  ·**Unclear** |
| **是否选择性报告结果** | ·**Yes高风险**不是所有的预先说明的首要结局均被报道 ；一个或多个首要结局为采用预先说明的测量方法、分析方法或数据子集来报道；系统评价感兴趣的一个或多个首要结局报道不全，以至于不能纳入meta分析；  ·**No 低风险**发表的报告包括所有的结局，包括预先说明的结局；  ·**Unsure** |
| **是否存在其它偏倚** | 具有与特殊试验设计相关的潜在偏倚来源，或被指欺诈， 或其它问题：否 |
| Notes  **备注** | 无 |

## 注意： 对方法学资料除“Yes”、“No”、“Unclear”选项外，还应 提示评价者尽可能引用原文所提供的语句，要尽可能对一些重要细 节进行详细描述。

## Results结果

Comparison:	一是进行的是什么样的比较，二是每一比较中使用的结局指标有哪些，三是结果资料的类型。

Outcome（结果数据）:

Subcategory（亚组）:

主要结局指标、次要结局指标

1、**神经阻滞的起效时间**（即各神经分支相应区域的针刺疼痛从注射完成到完全消失的时间）：尺侧阻滞起效时间 p＜0.05；其他神经（除尺N）阻滞起效时间 p＞0.05

2、**阻滞维持时间**（疼痛消失—有意识的伤口疼痛） p＜0.05（F=5.302,P=0.016）

|  | **A组（0.3%）**  **32人** | **B组（0.4%）**  **35人** | **C组（0.5%）**  **30人** |
| --- | --- | --- | --- |
| **阻滞维持时间（h）** | 2.2 ± 0.3 | 2.5 ± 0.6 | 2.6 ± 0.7 |

3、**The effective rate of anesthesia** p＜0.05

（Excellent表示：手术开始时不需要额外的镇静或镇痛药物

Effective表示：手术开始时出现疼痛或坐立不安，需补充镇静或镇痛药物继续手术

Invalid表示：手术开始时疼痛或坐立不安明显，即使加镇静或镇痛药也不能进行手术，最后使用全麻）

4、**麻醉并发症发生率**p＞0.05

|  | **A组（0.3%罗哌）**  **32人** | **B组（0.4%罗哌）**  **35人** | **C组（0.5%罗哌）**  **30人** |
| --- | --- | --- | --- |
| **并发症** | 血管神经损伤1例，鞘内意外注射1例，恶心呕吐1例，穿刺部位血肿明显1例 | 血压异常1例，鞘内意外注射1例，恶心呕吐1例，穿刺部位血肿明显1例 | 局麻药中毒1例，血气胸1例，恶心呕吐1例，穿刺部位血肿明显1例 |
| **发生率** | 12.5% | 11.4% | 13.3% |
| χ^2^=0.055, P=0.973 | | | |

**提取混杂因素：**

基金来源? 未说明

作者得出的关键性结论? 0.4% Ropivacaine is optimal.

作者对混杂因素的评价等? 无
