# Supplementary material for: Optimal concentration of ropivacaine for brachial plexus blocks in adult patients undergoing upper limb surgeries: a systematic review and meta-analysis
Source: Front Pharmacol. 2023 Nov 16;14:1288697. doi: 10.3389/fphar.2023.1288697 (PMC10687368; doi:10.3389/fphar.2023.1288697)
Supplement: Supplementary file 3 [file DataSheet1.ZIP › 锁骨上入路 3/0.5%罗哌/Ranjan R Venkatesh2016📕A Randomized Controlled Study of 0.5% Bupivacaine, 0.5% Ropivacaine and 0.75% Ropivacaine for Supraclavicular Brachial Plexus Block.doc]

**附件二：**

**基本信息**

**Date（提取日期）	Reviewer（提取者姓名）: 2023.01.11 吴琳**

**Study Title（文献标题）A Randomized Controlled Study of 0.5% Bupivacaine, 0.5% Ropivacaine and 0.75% Ropivacaine for Supraclavicular Brachial Plexus Block**

| First author**第一作者** | Ranjan R Venkatesh |
| --- | --- |
| Year of publication **出版年** | 2016 |
| Country of publication **出版国家** | India |
| Publication type**出版的类型** | Journal |
| **作者联系方式**  （一些文献能够获取的信息不全，需要联系作者） | ranjanrv2005@gmail.com |

**纳入文**献的合格性

| **研究是否为随机对照** | 是： ✔ 不是： |
| --- | --- |
| **参与者是否为诊断明确的患者** | ✔ |
| **干预措施是否符合要求** | 是：_✔__；不是：_______ |
| **备注：____________________________________________________________________________**  （如果研究不是随机对照干预文献即排除，注明排除的理由就好） | |

**研究对象的特征（参与者的总人数及各组人 数、人口学信息（性别、年龄等）、研究地点、纳入 人群的基础状况、有无合并症，以及纳入排除标准 等**）

| 研究对象的特征 | | Study |
| --- | --- | --- |
| **参与者的总人数及干预和对照组的各组人 数** | | 90人，30人/组  （1）Group A ：0.5% bupivacaine  （2）Group B ：0.5% ropivacaine  （3）Group C ：0.75% ropivacaine |
| **研究对象** | | - 2010年11月-2012年6月入组，拟在锁骨上入路BPB下行上肢手术的患者 - 各组对象基线一致（p＞0.05）   纳入对象基线表（如下表）    围手术期间：心率、血压、氧饱 p＞0.05 |
| **研究对象纳入标准** | | 1、 年龄 18-60岁 2、 ASA I-II级 3、 拟在锁骨上入路BPB下行上肢手术 |
| **研究对象排除标准** | | 1、 对局麻药过敏或有其他反应 2、 操作部位感染 3、 凝血功能障碍 4、 神经病史或精神疾病 5、 手术对侧半膈肌麻痹 6、 有活动性肝、肾疾病或慢性肾病史 7、 接受日间手术的病人 |
| **参与者的国家/研究地点** | | 来自印度的研究，在拟在锁骨上入路BPB下行上肢手术的患者 |
| **干预组的措施**  (包括持续的时间) | | （1）30ml/组/人。  （2）阻滞实施后，每4mins评估一次  ①运动阻滞：  -评估肩部（上束）-嘱患者抬高手臂，同时保持肘部伸直；  -评估手部（中束和下束）-通过握力判断  0-运动无减弱  1-轻度或部分瘫痪  2-瘫痪  ②感觉阻滞：针刺C5-T2区，分3级  0-针刺感觉未消失  1-镇痛（有触觉但无痛觉）  2-麻醉（无触觉、无痛觉）  （3）阻滞失败：阻滞后30mins肩外展仍未消失（5名患者，A组3人，B组1人，C组1人） |
| **对照组的措施**  (包括持续的时间) | |  |
| **随访时间** | | 无 |
| **结果数据** | **测量结果** | 见后面图表 |

## 方法Methods: Trial Quality：随机方案的产生，分配方案的隐 藏，是否使用盲法，是否存在结果数据的不完整，是 否选择性报告结果，及是否存在其它偏倚

| **随机对照数字的产生**  （对于分配序列的生成尽可能详细地说明分配序列的生成方法） | **RCT**  **-低风险**：计算机随机数字生成器 ，扔硬币，洗牌的卡片和信封，掷骰子，抽签等  **-高风险**：通过奇偶或出生日期产生序列 ，通过入院日期产生序列等  **- Unclear**  **Block randomization was done according to the computer generated random numbers generated using Microsoft Excel Program.** |
| --- | --- |
| Allocation concealment  **分配方案的隐藏**  （分配隐藏尽可能详细地描述隐藏随机分配序列的方法，并陈述隐藏 分配序列的可信度） | **·Yes** 低风险 第三方中心分配，顺序编号、不透明、密封的信封等  ·**Unclear**  **·No** 高风险 使用摊开的随机分配表（如随机序列清单），交替或循环，出生日期等**The random numbers were kept concealed in opaque sealed envelopes and were opened after obtaining informed consent from the patient.** |
| Blinding  **是否使用盲法**  因参与者和实施者了解干预情况而导致实施偏倚 | - Patients Yes No Not stated - Manipulator Yes No Not stated - Outcomes collectors Yes No Not stated - Data analysts Yes No Not stated   **The envelope was opened by an anaesthesiologist not involved in the study and the study medication was prepared and handed over to the researcher.**  **Neither the researcher nor the patient was aware of the study medication.**  **Immediately after the block placement, patients were evaluated every 4min，by an operator unaware of the injected solution** |
| Outcome assessment and measurement  **结果数据的完整性** | **·Yes低风险**：无缺失数据；缺失数据的数目在各干预组相当且各组缺失原因类似；  对二分类变量，与观察事件的发生风险相比，缺失比例不足以影响预估的干预效应；  对连续性结局数据，缺失数据的合理效应规模（均数差或标准均数差）不会大到影响观察的效应规模;  **·No** **高风险**：缺失数据的产生很大可能与真实结局相关, 缺失数据的数目及缺失原因在各干预组相差较大  对二分类变量，与观察事件的发生风险相比，缺失比例足以影响预估的干预效应  对连续性结局数据，缺失数据的合理效应规模（均数差或标准均数差）足以影响观察的效应规模;  ·**Unclear** |
| **是否选择性报告结果** | ·**Yes高风险**不是所有的预先说明的首要结局均被报道 ；一个或多个首要结局为采用预先说明的测量方法、分析方法或数据子集来报道；系统评价感兴趣的一个或多个首要结局报道不全，以至于不能纳入meta分析；  ·**No 低风险**发表的报告包括所有的结局，包括预先说明的结局；  ·**Unsure** |
| **是否存在其它偏倚** | 具有与特殊试验设计相关的潜在偏倚来源，或被指欺诈， 或其它问题 否 |
| Notes  **备注** | 作者分析本文局限性  1、研究中使用的罗哌卡因剂量未基于患者的体重；由于是一个固定的剂量，故可能会改变本研究的结果。 |

## 注意： 对方法学资料除“Yes”、“No”、“Unclear”选项外，还应 提示评价者尽可能引用原文所提供的语句，要尽可能对一些重要细 节进行详细描述。

## Results结果

Comparison:	一是进行的是什 么样的比较，二是每一比较中使用的结局指标有哪些，三是结果资料的类型。

Outcome（结果数据）:

Subcategory（亚组）:

主要结局指标、次要结局指标

1、感觉+运动 阻滞起效时间	P＞0.05

| 起效时间 | 0.5% ropivacaine | 0.75% ropivacaine | p |
| --- | --- | --- | --- |
| 感觉（min） | 17.79±5.03 | 18.48±6.14 | P＞0.05 |
| 运动（min） | 22.23±4.05 | 22.33±5.17 | P＞0.05 |

2、感觉+运动 阻滞持续时间 P＞0.05

| 持续时间 | 0.5% ropivacaine | 0.75% ropivacaine | p |
| --- | --- | --- | --- |
| 感觉（h） | 9.02±0.98 | 8.87±2.65 | P＞0.05 |
| 运动（h） | 8.29±0.92 | 7.89±2.74 | P＞0.05 |

Note：

提取混杂因素：

基金来源? 未说明

作者得出的关键性结论 **增加罗哌卡因的浓度（从0.5%到0.75%）未能改善阻滞起效时间和持续时间。因此，0.5%罗哌卡因似乎是一个更好的选择。**

作者对混杂因素的评价等? 未说明
