# Supplementary material for: Optimal concentration of ropivacaine for brachial plexus blocks in adult patients undergoing upper limb surgeries: a systematic review and meta-analysis
Source: Front Pharmacol. 2023 Nov 16;14:1288697. doi: 10.3389/fphar.2023.1288697 (PMC10687368; doi:10.3389/fphar.2023.1288697)
Supplement: Supplementary file 3 [file DataSheet1.ZIP › 锁骨上入路 3/Anitha Nileshwar2020📕Assessment of differential brachial plexus block using ropivacaine in intraoperative conscious patients A randomized control trial.doc]

**附件二：**

**基本信息**

**Date（提取日期）	Reviewer（提取者姓名）: 2023.01.13 吴琳**

**Study Title（文献标题）Assessment of differential brachial plexus block using ropivacaine in intraoperative conscious patients: A randomized control trial**

| First author**第一作者** | Anitha Nileshwar |
| --- | --- |
| Year of publication **出版年** | 2020 |
| Country of publication **出版国家** | India |
| Publication type**出版的类型** | Journal |
| **作者联系方式**  （一些文献能够获取的信息不全，需要联系作者） | kordcal85@gmail.com |

**纳入文**献的合格性

| **研究是否为随机对照** | 是：✔ 不是： |
| --- | --- |
| **参与者是否为诊断明确的患者** | ✔ |
| **干预措施是否符合要求** | 是：_✔__；不是：_______ |
| **备注：____________________________________________________________________________**  （如果研究不是随机对照干预文献即排除，注明排除的理由就好） | |

**研究对象的特征（参与者的总人数及各组人 数、人口学信息（性别、年龄等）、研究地点、纳入 人群的基础状况、有无合并症，以及纳入排除标准 等**）

| 研究对象的特征 | | Study |
| --- | --- | --- |
| **参与者的总人数及干预和对照组的各组人 数** | | 40人，2组  （1）0.15% ropivacaine 25ml  （2）0.1285% ropivacaine 25ml |
| **研究对象** | | - 2016.12-2018.6入组，拟在锁骨上入路臂丛神经阻滞下行手部或前臂手术的患者 - 各组对象基线一致（p＞0.05）   纳入对象基线表（如下表）   |
| **研究对象纳入标准** | | 1、 年龄 18-60岁 2、 ASA I-II级 3、 拟在锁骨上入路臂丛神经阻滞下行手部或前臂手术 4、 知情同意 |
| **研究对象排除标准** | | 1、 不愿意在手术期间保持清醒的患者 2、 已知困难气道 3、 已知对罗哌卡因过敏 |
| **参与者的国家/研究地点** | | 来自印度的研究，拟在锁骨上入路臂丛神经阻滞下行手部或前臂手术的患者 |
| **干预组的措施**  (包括持续的时间) | | （1）25ml/组/人。  （2）感觉阻滞评估：在小指(尺神经)、鱼际隆起(正中神经)、手背掌指关节(桡神经)和前臂外侧外侧(肌皮神经)采用针刺试验评估感觉阻滞。  （3）运动阻滞评估：运动功能为经拇指外展(桡神经)评估，拇指对位(正中神经)，拇指内收(尺神经)和屈曲肘部(肌皮神经)。   |
| **对照组的措施**  (包括持续的时间) | |  |
| **随访时间** | | 无 |
| **结果数据** | **测量结果** | 见后面图表 |

## 方法Methods: Trial Quality：随机方案的产生，分配方案的隐 藏，是否使用盲法，是否存在结果数据的不完整，是 否选择性报告结果，及是否存在其它偏倚

| **随机对照数字的产生**  （对于分配序列的生成尽可能详细地说明分配序列的生成方法） | **RCT**  **-低风险**：计算机随机数字生成器 ，扔硬币，洗牌的卡片和信封，掷骰子，抽签等  **-高风险**：通过奇偶或出生日期产生序列 ，通过入院日期产生序列等  **- Unclear**  **The patients were randomly allocated using computer-generated random number sequence into one of two groups.** |
| --- | --- |
| Allocation concealment  **分配方案的隐藏**  （分配隐藏尽可能详细地描述隐藏随机分配序列的方法，并陈述隐藏 分配序列的可信度） | **·Yes** 低风险 第三方中心分配，顺序编号、不透明、密封的信封等  ·**Unclear**  **·No** 高风险 使用摊开的随机分配表（如随机序列清单），交替或循环，出生日期等  **Allocation concealment was done by Serially Numbered Opaque Sealed Envelope (SNOSE) method.** |
| Blinding  **是否使用盲法**  因参与者和实施者了解干预情况而导致实施偏倚 | - Patients Yes No Not stated - Manipulator Yes No Not stated - Outcomes collectors Yes No Not stated - Data analysts Yes No Not stated   **All patients were blinded to the group assignment.**  **Anaesthesia Resident，who was blinded to the group assigned, serially assessed the patients after administration of the block.**  **Anaesthesia Consultant loaded the drugs and performed supraclavicular brachial plexus block, after receiving the allocation sequence from the independent statistician.** |
| Outcome assessment and measurement  **结果数据的完整性** | **·Yes低风险**：无缺失数据；缺失数据的数目在各干预组相当且各组缺失原因类似；  对二分类变量，与观察事件的发生风险相比，缺失比例不足以影响预估的干预效应；  对连续性结局数据，缺失数据的合理效应规模（均数差或标准均数差）不会大到影响观察的效应规模;  **·No** **高风险**：缺失数据的产生很大可能与真实结局相关, 缺失数据的数目及缺失原因在各干预组相差较大  对二分类变量，与观察事件的发生风险相比，缺失比例足以影响预估的干预效应  对连续性结局数据，缺失数据的合理效应规模（均数差或标准均数差）足以影响观察的效应规模;  ·**Unclear** |
| **是否选择性报告结果** | ·**Yes高风险**不是所有的预先说明的首要结局均被报道 ；一个或多个首要结局为采用预先说明的测量方法、分析方法或数据子集来报道；系统评价感兴趣的一个或多个首要结局报道不全，以至于不能纳入meta分析；  ·**No 低风险**发表的报告包括所有的结局，包括预先说明的结局；  ·**Unsure** |
| **是否存在其它偏倚** | 具有与特殊试验设计相关的潜在偏倚来源，或被指欺诈， 或其它问题 无 |
| Notes  **备注** | 无 |

## 注意： 对方法学资料除“Yes”、“No”、“Unclear”选项外，还应 提示评价者尽可能引用原文所提供的语句，要尽可能对一些重要细 节进行详细描述。

## Results结果

Comparison:	一是进行的是什 么样的比较，二是每一比较中使用的结局指标有哪些，三是结果资料的类型。

Outcome（结果数据）:

Subcategory（亚组）:

主要结局指标、次要结局指标

1、感觉阻滞起效、持续时间

|  | 0.15% ropivacaine | 0.1285% ropivacaine | p |
| --- | --- | --- | --- |
| 感觉阻滞起效时间 | 20±3.73（mins） | 19±4.59（mins） | p=0.531 |
| 切口阻滞持续时间 | 20±3.24（mins） | 19.5±3.2（mins） | p=0.627 |

2、镇痛药需要率、需转全麻率

|  | 0.15% ropivacaine | 0.1285% ropivacaine | p |
| --- | --- | --- | --- |
| 镇痛药需要率 | 6/20（30%） | 14/20（70%） | p=0.011 |
| 需转全麻率 | 0/20（0%） | 8/20（40%） | p＜0.05 |

3、患者阻滞满意度

|  | 0.15% ropivacaine | 0.1285% ropivacaine | p |
| --- | --- | --- | --- |
| 阻滞满意度 | 2（IQR 0-2） | 2（IQR 2-6） | p=0.021 |

4、并发症

|  | 0.15% ropivacaine | 0.1285% ropivacaine | p |
| --- | --- | --- | --- |
| 并发症 | 0/20（0%） | 0/20（0%） | p＞0.05 |

Note：

提取混杂因素：

基金来源? 未提及

作者得出的关键性结论? Differential block sufficient to permit surgery of short duration is possible with both concentrations **but success rate is higher with 0.15% ropivacaine**.（ 0.15%罗哌卡因成功率更高）

作者对混杂因素的评价等? 无
