# Supplementary material for: Optimal concentration of ropivacaine for brachial plexus blocks in adult patients undergoing upper limb surgeries: a systematic review and meta-analysis
Source: Front Pharmacol. 2023 Nov 16;14:1288697. doi: 10.3389/fphar.2023.1288697 (PMC10687368; doi:10.3389/fphar.2023.1288697)
Supplement: Supplementary file 3 [file DataSheet1.ZIP › 锁骨上入路 3/G. Fang2016📕The Minimum Effective Concentration of Ropivacaine in Ultrasound-guided Supraclavicular Block.doc]

**附件二：**

**基本信息**

**Date（提取日期）	Reviewer（提取者姓名）: 2023.01.14 吴琳**

**Study Title（文献标题）The Minimum Effective Concentration of Ropivacaine in Ultrasound-guided Supraclavicular Block**

| First author**第一作者** | G. Fang |
| --- | --- |
| Year of publication **出版年** | 2016 |
| Country of publication **出版国家** | China |
| Publication type**出版的类型** | Journal |
| **作者联系方式**  （一些文献能够获取的信息不全，需要联系作者） | alluo@tjh.tjmu.edu.cn |

**纳入文**献的合格性

| **研究是否为随机对照** | 是：✔ 不是： |
| --- | --- |
| **参与者是否为诊断明确的患者** | ✔ |
| **干预措施是否符合要求** | 是：_✔__；不是：_______ |
| **备注：____________________________________________________________________________**  （如果研究不是随机对照干预文献即排除，注明排除的理由就好） | |

**研究对象的特征（参与者的总人数及各组人 数、人口学信息（性别、年龄等）、研究地点、纳入 人群的基础状况、有无合并症，以及纳入排除标准 等**）

| 研究对象的特征 | | Study |
| --- | --- | --- |
| **参与者的总人数及干预和对照组的各组人 数** | | **51**人。 |
| **研究对象** | | - 2014.11-2015.03入组，拟在SCB下行单侧上肢手术的患者 - 各组对象基线一致（p＞0.05）   纳入对象基线表（如下表）   |
| **研究对象纳入标准** | | 1、 年龄 41-65岁 2、 ASA I-III级 3、 BMI 18-35 kg.m^-2^ |
| **研究对象排除标准** | | 1、 无法知情同意 2、 孕妇 3、 对局麻药过敏 4、 既往有神经病变，凝血功能障碍和既往锁骨上区手术史。 |
| **参与者的国家/研究地点** | | 如来自美国的研究，在 社区照顾中心或家庭中生活的老年人 |
| **干预组的措施**  (包括持续的时间) | | （1）40ml/组/人。  （2）阻滞评估：SCB后每10mins/次，直至30mins。  -感觉阻滞分级（3级）：与对侧相比（肌皮神经，桡神经，正中神经和尺神经）  0-无阻滞；1-不完全阻滞；2-完全阻滞  阻滞成功的条件：在30min内4条神经感觉完全阻滞，并且患者进行了无痛手术，不需要补充阿片类药物、全身麻醉或局部浸润麻醉 |
| **对照组的措施**  (包括持续的时间) | |  |
| **随访时间** | | 术后一周（并发症情况） |
| **结果数据** | **测量结果** | 见后面图表 |

## 方法Methods: Trial Quality：随机方案的产生，分配方案的隐 藏，是否使用盲法，是否存在结果数据的不完整，是 否选择性报告结果，及是否存在其它偏倚

| **随机对照数字的产生**  （对于分配序列的生成尽可能详细地说明分配序列的生成方法） | **RCT**  **-低风险**：计算机随机数字生成器 ，扔硬币，洗牌的卡片和信封，掷骰子，抽签等  **-高风险**：通过奇偶或出生日期产生序列 ，通过入院日期产生序列等  **- Unclear**  The envelopes were prepared by a resident who took no further part in the study. |
| --- | --- |
| Allocation concealment  **分配方案的隐藏**  （分配隐藏尽可能详细地描述隐藏随机分配序列的方法，并陈述隐藏 分配序列的可信度） | **·Yes** 低风险 第三方中心分配，顺序编号、不透明、密封的信封等  ·**Unclear**  **·No** 高风险 使用摊开的随机分配表（如随机序列清单），交替或循环，出生日期等  Thus, we prospectively recruited patients until 45 successful blocks were accomplished, and a set of 44 sealed envelopes (with the random concentration assignments inside for successful blocks) were opened. |
| Blinding  **是否使用盲法**  因参与者和实施者了解干预情况而导致实施偏倚 | - Patients Yes No Not stated - Manipulator Yes No Not stated - Outcomes collectors Yes No Not stated - Data analysts Yes No Not stated   Subsequently, brachial plexus blockade was assessed by a single observer (L), blinded to the ropivacaine concentration used, every 10 min until 30 min after completion of the injection. |
| Outcome assessment and measurement  **结果数据的完整性** | **·Yes低风险**：无缺失数据；缺失数据的数目在各干预组相当且各组缺失原因类似；  对二分类变量，与观察事件的发生风险相比，缺失比例不足以影响预估的干预效应；  对连续性结局数据，缺失数据的合理效应规模（均数差或标准均数差）不会大到影响观察的效应规模;  **·No** **高风险**：缺失数据的产生很大可能与真实结局相关, 缺失数据的数目及缺失原因在各干预组相差较大  对二分类变量，与观察事件的发生风险相比，缺失比例足以影响预估的干预效应  对连续性结局数据，缺失数据的合理效应规模（均数差或标准均数差）足以影响观察的效应规模;  ·**Unclear** |
| **是否选择性报告结果** | ·**Yes高风险**不是所有的预先说明的首要结局均被报道 ；一个或多个首要结局为采用预先说明的测量方法、分析方法或数据子集来报道；系统评价感兴趣的一个或多个首要结局报道不全，以至于不能纳入meta分析；  ·**No 低风险**发表的报告包括所有的结局，包括预先说明的结局；  ·**Unsure** |
| **是否存在其它偏倚** | 具有与特殊试验设计相关的潜在偏倚来源，或被指欺诈， 或其它问题 否 |
| Notes  **备注** | 无 |

## 注意： 对方法学资料除“Yes”、“No”、“Unclear”选项外，还应 提示评价者尽可能引用原文所提供的语句，要尽可能对一些重要细 节进行详细描述。

## Results结果

Comparison:	一是进行的是什 么样的比较，二是每一比较中使用的结局指标有哪些，三是结果资料的类型。

Outcome（结果数据）:

Subcategory（亚组）:

主要结局指标、次要结局指标

1、**The MEC90 was 0.257% (95% CI 0.241–0.280%)**

the MEC95 was estimated to be 0.266% (95% CI 0.255–0.282%);

the MEC99 was 0.273% (95% CI 0.266–0.282%)

2、阻滞成功率

3、并发症

Note：

提取混杂因素：

基金来源? 未说明

作者得出的关键性结论? The minimum effective ropivacaine concentration in 90% of subjects was **0.257% ropivacaine.**

作者对混杂因素的评价等? 无
